# Integration of habitat radiomics and traditional radiomic features for predicting pathological complete response in esophageal squamous cell carcinoma following neoadjuvant immunotherapy and chemotherapy: a multicenter comparative study

**DOI:** 10.1186/s12967-025-07522-y

**Published:** 2026-01-13

**Authors:** Zhiyun Xu, Yijiang Lu, Fengyi Zuo, Hanlin Ding, Yipeng Feng, Xiaokang Shen, Xuming Song, Wenjie Xia, Qixing Mao, Bing Chen, Rutao Li, Hui Wang, Lin Xu, Gaochao Dong, Feng Jiang

**Affiliations:** 1 Department of Thoracic Surgery, Nanjing Medical University Affiliated Cancer Hospital & Jiangsu Cancer Hospital & Jiangsu Institute of Cancer Research, Nanjing, P. R. China; 2 Jiangsu Key Laboratory of Molecular and Translational Cancer Research, Cancer Institute of Jiangsu Province, Nanjing, P. R. China; 3 The Fourth Clinical College of Nanjing Medical University, Nanjing, P. R. China; 4 Department of Thoracic Surgery, The Affiliated Huai’an No. 1 People’s Hospital of Nanjing Medical University, Huai’an, P. R. China; 5 Department of Thoracic and Cardiovascular Surgery, Nanjing First Hospital, Nanjing Medical University, Nanjing, P. R. China; 6 Department of Thoracic Surgery, The Fourth Affiliated Hospital of Soochow University, Soochow, P. R. China; 7 The Second Clinical Medical School of Nanjing Medical University, Nanjing, P. R. China

**Keywords:** Esophageal squamous cell carcinoma, Pathological complete response, Tumor heterogeneity, Habitat radiomics, Multicenter study

## Abstract

**Background:**

Esophageal squamous cell carcinoma (ESCC) remains one of the leading causes of cancer-related mortality worldwide. Although immunotherapy has shown promising efficacy for locally advanced ESCC, the lack of reliable predictive tools and the marked heterogeneity of tumors make it difficult to accurately evaluate treatment responses. To address this challenge, we conducted a multicenter study aimed at developing and comparing predictive models based on habitat radiomics and traditional radiomic features to estimate pathological complete response (pCR) in patients receiving neoadjuvant immunotherapy and chemotherapy. Using multicenter data, we systematically assessed the performance of these models to determine the relative advantages of each feature type in predicting treatment outcomes and supporting personalized therapeutic strategies.

**Methods:**

This retrospective study analyzed ESCC patient data from three medical centers. Pre-treatment CT imaging was utilized for tumor region segmentation and the extraction of both Habitat Radiomics and traditional Radiomic Features. Feature selection was performed using LASSO regression, and machine learning models were developed based on these features. Several machine learning algorithms, including Support Vector Machines (SVM), Random Forest, and XGBoost, were employed for training and validation. Model performance was evaluated using metrics such as ROC curves, AUC, sensitivity, and specificity.

**Results:**

The Habitat Radiomics model achieved AUCs of 0.938 in the training cohort, 0.896 in the internal validation cohort, 0.819 in external validation cohort 1, and 0.846 in external validation cohort 2, demonstrating strong and consistent predictive performance. In comparison, the traditional Radiomics model yielded AUCs of 0.941, 0.845, 0.796, and 0.729, respectively. Beyond higher AUC values, the Habitat Radiomics model also showed superior sensitivity and specificity in predicting pCR. Notably, the combined model that integrated both Habitat and traditional Radiomics features outperformed the individual models, achieving the highest AUC of 0.960 across cohorts and underscoring its superior predictive accuracy.

**Conclusion:**

This study demonstrates that Habitat Radiomics features provide significant advantages over traditional Radiomics in predicting immunotherapy response in ESCC patients. The combined model, integrating both feature sets, shows exceptional predictive performance, with promising clinical applications in personalized treatment strategies. Future research will explore the broader applicability of this model across different cancer types and its integration with additional biomarkers to further enhance prediction accuracy.

**Supplementary Information:**

The online version contains supplementary material available at 10.1186/s12967-025-07522-y.

## Introduction

Esophageal squamous cell carcinoma (ESCC) remains a leading cause of cancer-related mortality worldwide, particularly in Asia, with high incidence and mortality rates [1–3]. Neoadjuvant immunotherapy combined with chemotherapy has shown promising efficacy for locally advanced ESCC, but predicting treatment response remains a significant challenge [4]. Immunotherapy, particularly immune checkpoint inhibitors targeting PD-1/PD-L1, has revolutionized cancer treatment; however, determining which patients will respond to treatment remains elusive [5–7]. Tumor heterogeneity, encompassing genetic, phenotypic, and microenvironmental variations, plays a crucial role in determining the effectiveness of immunotherapy [8]. The lack of reliable predictive biomarkers complicates the accurate evaluation of therapeutic outcomes, thus hindering personalized treatment strategies. Hence, developing robust predictive tools to assess immunotherapy response is essential for optimizing treatment and improving outcomes in ESCC patients.

Radiomics, a rapidly emerging field, offers a promising approach to predicting treatment outcomes by extracting quantitative features from medical imaging [9]. Traditional radiomics analyzes global tumor characteristics such as shape and texture and has been applied in various cancers, including lung, breast, and colorectal [10–13]. However, traditional radiomics fails to capture spatial heterogeneity within the tumor, which is critical for predicting responses to immunotherapy [14]. Habitat radiomics addresses this by dividing the tumor into spatially distinct regions, providing a more detailed characterization of tumor microenvironmental heterogeneity [15]. This approach enhances predictive potential for treatment response, particularly in immunotherapy.

Currently, pathological complete response (pCR) prediction in ESCC has mainly been explored using tissue- and blood-based biomarkers, including PD-L1 expression, tumor mutation burden (TMB), microsatellite instability, tumor-infiltrating lymphocytes, circulating tumor DNA, and systemic inflammatory indices, alongside conventional imaging such as CT scans [16, 17]. However, these biomarkers require invasive sampling and are vulnerable to spatial and temporal heterogeneity, and therefore may not fully reflect whole-tumor biology. In this study, non-invasive imaging biomarkers—particularly radiomics and habitat radiomics derived from pre-treatment CT—offer a promising complementary approach for preoperatively estimating the probability of pCR following neoadjuvant chemoimmunotherapy [18]. Furthermore, CT imaging, although useful for assessing tumor morphology, cannot fully capture the tumor microenvironment’s complexity or its influence on immunotherapy responses [19]. In contrast, habitat radiomics offers a more detailed, objective, and reproducible method, providing deeper insights into tumor subregions that directly influence treatment response [20].

The primary innovation of this study lies in the direct comparison of habitat radiomics and traditional radiomics for predicting pCR in ESCC patients undergoing neoadjuvant immunotherapy combined with chemotherapy. While prior research has explored traditional radiomics, few studies have focused on habitat radiomics for capturing tumor heterogeneity in immunotherapy contexts. Our study uses multicenter data, ensuring broader applicability and generalizability, and integrates both habitat and traditional radiomics into a combined model to improve predictive accuracy. This combined approach enhances our understanding of tumor biology and provides a more reliable tool for predicting immunotherapy response.

The objective of this study is to compare the predictive performance of habitat and traditional radiomics features in forecasting pCR following neoadjuvant immunotherapy and chemotherapy in ESCC patients. We aim to construct and validate machine learning models incorporating both feature types and assess their advantages in predicting treatment response. Additionally, we will develop a nomogram based on these models to provide a clinical decision-support tool for personalized treatment planning. We hypothesize that habitat radiomics will improve pCR prediction accuracy by better capturing tumor heterogeneity and the tumor microenvironment. We expect that a combined model will outperform individual models, offering a more reliable tool for clinical decision-making in personalized ESCC treatment.

## Materials and methods

### Patient selection and data collection

**Fig. 1:**
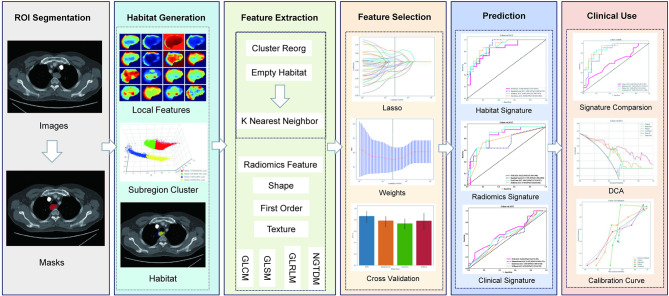
Patient selection process across three medical centers. Flowchart depicting the patient inclusion process across three medical centers. Center 1, The Affiliated Huaian No. 1 People’s Hospital of Nanjing Medical University; Center 2, The Affiliated Cancer Hospital of Nanjing Medical University; Center 3, Nanjing First Hospital

This retrospective study was conducted across three medical centers: the Affiliated Huai’an No. 1 People’s Hospital of Nanjing Medical University, the Affiliated Cancer Hospital of Nanjing Medical University, and Nanjing First Hospital, as illustrated in the flowchart shown in Fig. 1. Eligible patients were those with histopathologically confirmed esophageal squamous cell carcinoma (ESCC) diagnosed via endoscopic biopsy, clinically staged as locally advanced (cT1N1–T3N0–3M0) according to contrast-enhanced computed tomography (CT) findings and the 8th edition of the American Joint Committee on Cancer (AJCC) staging system. All patients completed a standardized neoadjuvant chemoimmunotherapy (NACIT) regimen prior to surgery, consisting of two cycles of camrelizumab in combination with albumin-bound paclitaxel and cisplatin administered at fixed intervals before resection. The same NACIT protocol was adopted across the three participating centers, and patients who received non-standard regimens or additional systemic agents were excluded to minimize treatment-related heterogeneity. Additional requirements included the availability of a high-quality contrast-enhanced chest CT scan in the venous phase with a slice thickness of ≤ 5 mm and without significant artifacts, performed within 14 days before initiating NACIT, and the completion of R0 resection within 6–8 weeks after treatment. Only patients aged 18 years or older and with complete postoperative pathological reports documenting pathological response status (pCR or non-pCR) were included. Patients were excluded if they had histologic subtypes other than squamous cell carcinoma, had received radiotherapy, targeted therapy, or immunotherapy without concurrent neoadjuvant chemotherapy, or presented with distant metastases (M1) or synchronous malignancies at baseline. Those with incomplete clinical data or missing key endpoints, such as absent pathological response information or loss to follow-up before surgery, were also excluded. Imaging-related exclusion criteria comprised poor-quality or incomplete CT scans, absence of venous-phase enhancement, severe motion artifacts, tumor invisibility, or acquisition parameters inconsistent with study requirements (slice thickness > 5 mm or lack of intravenous contrast). Patients with an interval exceeding 14 days between imaging and NACIT initiation, tumors deemed unresectable or patients considered unfit for surgery after NACIT by a multidisciplinary team, as well as those who received non-standard treatment regimens or were concurrently enrolled in other interventional protocols, were also excluded. As shown in Fig. 1, a total of 336 patients were initially identified, and after applying the eligibility criteria described above, all 336 patients met the requirements for final analysis. These patients were randomly allocated into three cohorts: 101 in the training cohort, 43 in the internal validation cohort, and 117 and 75 in two external test cohorts. Clinical variables collected for analysis included age, sex, body mass index (BMI), smoking history, hypertension, diabetes, and surgical parameters such as operative duration and intraoperative blood loss. This retrospective study was approved by the Institutional Review Board (IRB) of the Affiliated Huai’an No. 1 People’s Hospital of Nanjing Medical University (Approval No: KY-2024-373-01). The requirement for informed consent was waived due to the retrospective nature of the study, and all patient data were anonymized to protect confidentiality.

### Imaging data acquisition and preprocessing

**Fig. 2:**
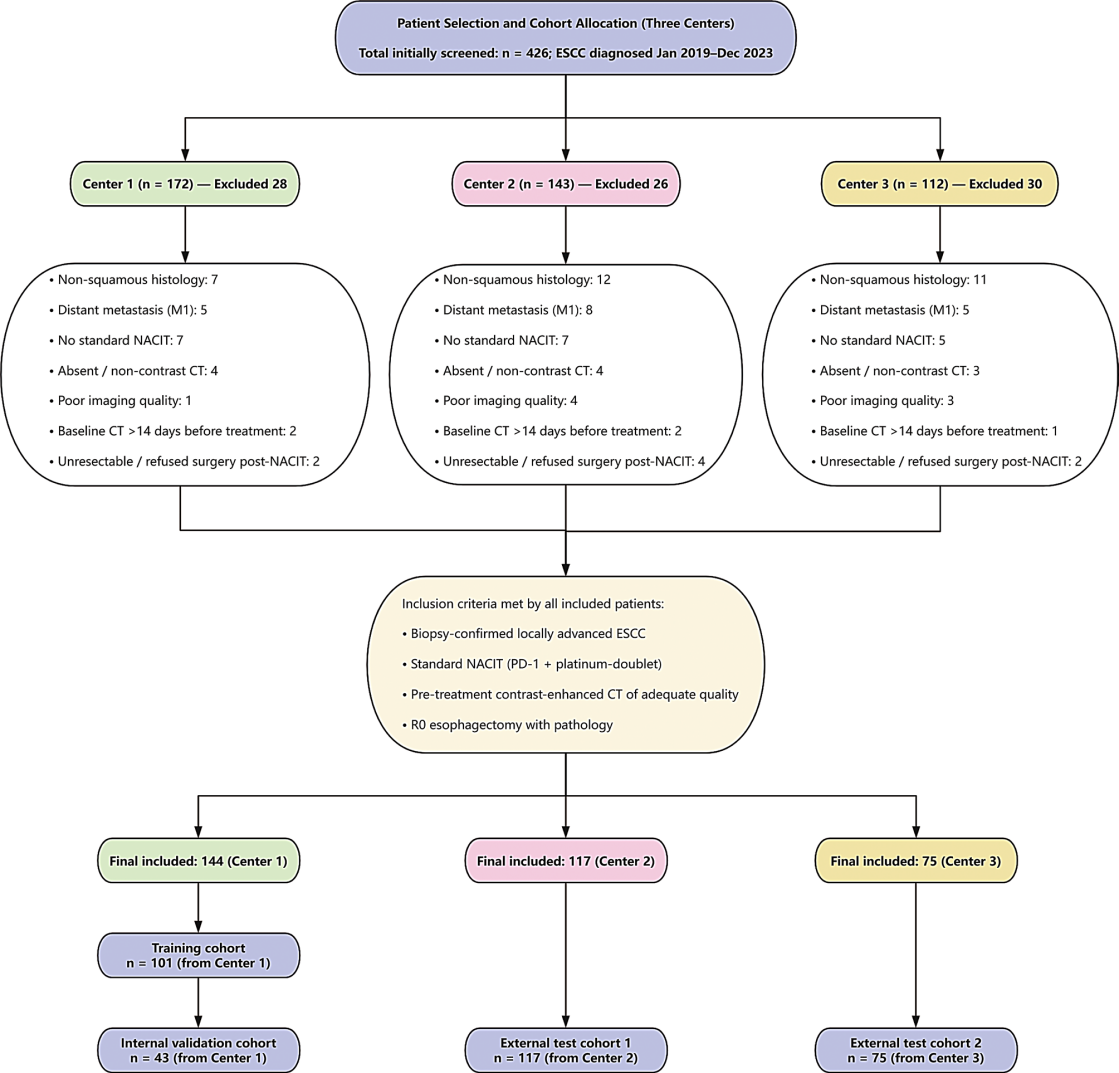
Overall study workflow. The schematic illustrates the full analytical pipeline, beginning with pretreatment CT imaging acquisition and tumor segmentation, followed by voxel-level feature extraction, habitat clustering, radiomic feature extraction, feature selection, and multi-model construction (Habitat, Radiomics, Clinical, Combined). The final steps involve model evaluation and visualization through ROC, calibration, DCA, and nomogram. Each phase is structured to predict pathological complete response (pCR) after neoadjuvant chemoimmunotherapy (NACIT) in locally advanced ESCC

As shown in Fig. 2, CT imaging was performed for all patients using a standardized protocol across the three medical centers. Pre-treatment contrast-enhanced CT images were acquired with slice thicknesses ranging from 1 to 5 mm. Tumor segmentation was performed manually by experienced radiologists using specialized software to ensure accurate delineation of the tumor regions. The images were resampled to a uniform resolution of 1 mm × 1 mm × 1 mm to maintain consistency across patients and imaging platforms. To address inter-scanner and inter-institution variability, image harmonization was performed using the ComBat normalization method to reduce batch effects from different CT machines. This approach ensured that imaging data across different scanners and institutions could be integrated effectively. Tumor segmentation was carried out by two experienced radiologists, and consensus masks were used to minimize variability in the delineation process. To ensure image quality and consistency, intraclass correlation coefficients (ICC) were calculated for inter-rater reliability during tumor segmentation. The ICC was calculated across multiple radiologists to evaluate the consistency of tumor delineation, and a threshold of ICC ≥ 0.85 was considered acceptable. Preprocessing steps included noise reduction through Gaussian filtering to eliminate image noise, intensity normalization to a standard range (0–255) to minimize inter-patient variations, and consistent image reconstruction parameters to eliminate discrepancies across different CT scanners. These preprocessed images were then used for radiomic feature extraction.

### Radiomic feature extraction

Traditional radiomic features were extracted from the pre-treatment CT scans to characterize the overall tumor characteristics. These included first-order statistics, which quantify the intensity distribution of the tumor (e.g., mean, variance, skewness, kurtosis); texture features, which were extracted from the Gray-Level Co-occurrence Matrix (GLCM), Gray-Level Run Length Matrix (GLRLM), and Gray-Level Size Zone Matrix (GLSZM), and describe the spatial relationship between neighboring voxels to assess tumor heterogeneity; and shape features, which quantify geometric properties such as volume, surface area, and sphericity.

The features were extracted using the open-source PyRadiomics package, which implements standardized algorithms for radiomic feature extraction. For habitat radiomics, the tumor regions were first segmented by radiologists. Then, the K-means clustering algorithm was applied to partition the tumor into spatially distinct subregions (referred to as “habitats”), based on voxel-level features. Each habitat represented a region with unique imaging characteristics. Local texture features were then extracted from each identified habitat to describe the heterogeneity within these subregions, using the same set of texture matrices (GLCM, GLRLM, GLSZM), but computed separately for each habitat. This approach allowed for a deeper understanding of the tumor’s microenvironment, identifying regional variations that may influence treatment response.

### Feature selection and model training

To ensure that only the most relevant features were included in the predictive models, LASSO (Least Absolute Shrinkage and Selection Operator) regression was applied for feature selection. LASSO regression uses regularization to shrink the coefficients of less relevant features to zero, retaining only those features that provide significant predictive value. The optimal regularization parameter (λ) was determined using 10-fold cross-validation to minimize mean squared error (MSE). A total of 16 features were retained after feature selection. These features were standardized before training to ensure consistency across different scales.

Several machine learning algorithms were employed to construct predictive models based on the selected features. These included Support Vector Machines (SVM), a classifier that creates hyperplanes to separate different classes in high-dimensional space; Random Forest, an ensemble method that aggregates the outputs of multiple decision trees; and XGBoost, a gradient-boosting algorithm that iteratively builds trees to correct errors from previous iterations, improving predictive performance, particularly in imbalanced datasets.

Cross-validation was performed in a nested manner, where the LASSO feature selection process was conducted inside each fold of the cross-validation to avoid overfitting and ensure a robust evaluation of the model performance. Each model was trained using the training cohort and validated on the internal validation cohort to assess generalizability. Hyperparameters for each model were optimized using grid search and cross-validation. The search spaces were: max_iter = 1–100 and max_depth = 1–5 for applicable models; for SVM, we evaluated kernels = [linear, polynomial, RBF]. Other model-specific ranges showed minor differences across algorithms.

### Model performance evaluation

The performance of each model was evaluated using multiple metrics, including the Receiver Operating Characteristic (ROC) curve, to visualize the trade-off between sensitivity and specificity at various thresholds. Area Under the Curve (AUC) was calculated to quantify the discriminatory ability of the models, with higher AUC values indicating better performance. Sensitivity and Specificity were assessed to determine the model’s ability to correctly identify pCR and non-pCR cases. Positive Predictive Value (PPV) and Negative Predictive Value (NPV) were also calculated to evaluate the proportion of true positive and true negative predictions made by the model.

Additionally, calibration curves were generated to assess the alignment of predicted probabilities with observed outcomes. Models with accurate probabilistic predictions will demonstrate a close fit to the diagonal line. Decision Curve Analysis (DCA) was employed to evaluate the clinical utility of each model, measuring the net benefit of using the model across different threshold probabilities and guiding treatment decisions in clinical practice.

### Nomogram construction

To provide a user-friendly tool for clinical decision-making, a nomogram was constructed based on the combined radiomics model (integrating both habitat and traditional radiomics features) and clinical variables such as tumor stage and patient age. The nomogram allows clinicians to estimate the probability of a patient achieving pCR based on individual patient characteristics. Each variable is assigned a score according to its contribution to the overall risk, and the total score provides a personalized prediction of treatment outcome. This tool serves as a clinical decision support system to guide treatment planning, especially in selecting patients for neoadjuvant immunotherapy and chemotherapy.

### Statistical analysis

Descriptive statistics were employed to summarize patient characteristics, with continuous variables presented as mean ± standard deviation and categorical variables as frequencies and percentages. DeLong tests were conducted to compare the performance of the models, assessing differences in the area under the curve (AUC). Pairwise comparisons were used to determine statistical significance, with p-values < 0.05 considered statistically significant. The goodness-of-fit of logistic regression models was evaluated using the Hosmer-Lemeshow test. For model validation, performance metrics were computed for the training, internal validation, and external test cohorts. Cross-validation was applied to mitigate overfitting and ensure the generalizability of the models across diverse patient populations. All computational analyses were conducted using Python 3.7.12. Statistical tests were carried out using Statsmodels (v0.13.2), and radiomics features were extracted with PyRadiomics (v3.0.1). Machine learning algorithms, including support vector machines (SVM), were implemented using scikit-learn (v1.0.2). Deep learning models, where applicable, were developed in PyTorch (v1.11.0), supported by CUDA (v11.3.1) and cuDNN (v8.2.1) for GPU acceleration.

## Results

### Baseline characteristics

The baseline clinical characteristics of patients in the training, internal validation, and external test cohorts are summarized in Table 1. A total of 336 patients were included in the study, with 101 patients in the training cohort, 43 in the internal validation cohort, and 117 and 75 in the two external test cohorts. Clinical variables, including age, sex, body mass index (BMI), smoking history, comorbidities (hypertension, diabetes), and surgical data (operative duration, intraoperative blood loss), were collected and analyzed. Statistical analysis revealed no significant differences between the cohorts for all clinical variables (*p* > 0.05), suggesting that the patient populations across the training, validation, and test cohorts were well balanced. This homogeneity ensures the comparability of the cohorts for model performance evaluation, minimizing potential bias. Univariable and multivariable logistic regression analyses of clinical predictors for pCR are detailed in Table 2. Key findings from these analyses indicate that age, BMI, and FEV1/FVC% were not significantly associated with pCR (all *p* > 0.05). In contrast, left ventricular ejection fraction (LVEF) was significantly associated with pCR in univariable analysis (*p* < 0.05), though this association became marginal in multivariable analysis (*p* = 0.095). Furthermore, postoperative delirium was identified as a significant predictor of pCR in univariable analysis (*p* < 0.05), but the association was less pronounced in multivariable analysis (*p* = 0.09). Notably, tumor stage (cT) was strongly associated with pCR, with T2 and T3 tumors showing significantly higher odds of achieving pCR, as reflected by odds ratios of 1.392 (95% CI: 1.175–1.651) and 1.738 (95% CI: 1.456–2.074), respectively (both *p* < 0.001).

**Table 1:** Baseline clinical characteristics of patients across Training, internal Validation, and external test cohorts

Feature name	Train cohort (*n* = 101)	Validation cohort (*n* = 43)	Test1cohort (*n* = 117)	Test2 cohort (*n* = 75)	*P* value
Age (years)	65.13 ± 6.78	65.26 ± 5.37	65.29 ± 6.95	66.00 ± 5.89	0.911
Gender					1.0
Female	45 (44.55%)	19 (44.19%)	56 (47.86%)	33(44.00%)	
Male	56 (55.45%)	24 (55.81%)	61 (52.14%)	42 (56.00%)	
BMI (kg/m²)	22.70 ± 2.51	22.89 ± 2.22	22.57 ± 2.49	22.93 ± 2.34	0.641
FEV1(% predicted)	89.52 ± 17.65	94.89 ± 20.50	92.33 ± 19.72	94.20 ± 22.23	0.176
FEV1/FVC%	104.99 ± 12.01	103.77 ± 16.78	105.22 ± 11.74	105.96 ± 11.33	0.585
LVEF	65.69 ± 2.18	65.64 ± 2.56	66.27 ± 2.00	66.07 ± 2.24	0.935
Hypertension					0.446
No	52 (51.49%)	26 (60.47%)	59 (50.43%)	36 (48.00%)	
Yes	49 (48.51%)	17 (39.53%)	58 (49.57%)	39 (52.00%)	
Diabetes Mellitus					0.353
No	80 (79.21%)	38(88.37%)	99 (84.62%)	63 (84.00%)	
Yes	21 (20.79%)	5 (11.63%)	18 (15.38%)	12(16.00%)	
Coronary Heart Disease					0.068
No	60 (59.41%)	33 (76.74%)	73 (62.39%)	45 (60.00%)	
Yes	41 (40.59%)	10 (23.26%)	44 (37.61%)	30 (40.00%)	
Smoking					0.612
No	65 (64.36%)	30 (69.77%)	81 (69.23%)	44 (58.67%)	
Yes	36 (35.64%)	13 (30.23%)	36 (30.77%)	31 (41.33%)	
Drinking					0.511
No	66 (65.35%)	31 (72.09%)	79 (67.52%)	48 (64.00%)	
Yes	35 (34.65%)	12 (27.91%)	38 (32.48%)	27 (36.00%)	
ASA PG					0.091
I	27 (26.73%)	17 (39.53%)	34 (29.06%)	18 (24.00%)	
II	68 (67.33%)	26 (60.47%)	80 (68.38%)	56 (74.67%)	
III	6 (5.94%)	0 (0.00%)	3 (2.56%)	1 (1.33%)	
Operative duration (minutes)	271.94 ± 37.11	258.98 ± 36.16	273.47 ± 42.81	284.00 ± 48.49	0.058
Intraoperative blood loss (mL)	134.25 ± 62.73	137.23 ± 59.06	113.18 ± 46.54	123.27 ± 46.42	0.565
Tumor length (cm)	3.89 ± 0.68	3.74 ± 0.65	3.80 ± 0.66	3.81 ± 0.65	0.276
Total number of lymph nodes	20.92 ± 2.83	20.57 ± 2.47	20.83 ± 2.72	21.20 ± 2.62	0.485
Ki67	2.64 ± 1.48	2.47 ± 1.36	2.12 ± 1.42	2.56 ± 1.60	0.467
PNI	43.83 ± 7.88	41.37 ± 6.80	43.34 ± 6.92	43.08 ± 6.56	0.081
NLR	3.95 ± 2.16	4.39 ± 2.22	3.54 ± 1.95	4.17 ± 2.18	0.285
SII	835.97 ± 453.91	958.41 ± 443.44	835.51 ± 487.16	855.89 ± 401.22	0.058
Tumor location					0.943
Upper	24 (23.76%)	11 (25.58%)	32 (27.35%)	18 (24.00%)	
Middle	50 (49.50%)	20 (46.51%)	52 (44.44%)	43 (57.33%)	
Low	27 (26.73%)	12 (27.91%)	33 (28.21%)	14 (18.67%)	
cT					0.751
T1	8 (7.92%)	5 (11.63%)	6 (5.13%)	3 (4.00%)	
T2	35 (34.65%)	13 (30.23%)	41 (35.04%)	27 (36.00%)	
T3	58 (57.43%)	25 (58.14%)	70 (59.83%)	45 (60.00%)	
cN					0.876
N0	52 (51.49%)	21 (48.84%)	67 (57.26%)	35 (46.67%)	
N1	37 (36.63%)	17 (39.53%)	39 (33.33%)	31 (41.33%)	
N2	12 (11.88%)	5 (11.63%)	11 (9.40%)	9 (12.00%)	
cTNM					0.322
II	68 (67.33%)	25 (58.14%)	73 (62.39%)	41 (54.67%)	
III	33 (32.67%)	18 (41.86%)	44 (37.61%)	34 (45.33%)	
ypT					0.592
T0	27 (26.73%)	13 (30.23%)	37 (31.62%)	22 (28.00%)	
T1	36 (35.64%)	16 (37.21%)	43 (36.75%)	27 (36.00%)	
T2	24 (23.76%)	9 (20.93%)	25 (21.37%)	18 (24.00%)	
T3	14 (13.86%)	5 (11.63%)	12 (10.26%)	8 (10.67%)	
ypN					0.321
N0	74 (72.28%)	35 (81.40%)	99 (83.76%)	60 (80.00%)	
N1	19 (18.81%)	6 (13.95%)	15 (12.82%)	13 (17.33%)	
N2	8 (7.92%)	2 (4.65%)	5 (4.27%)	2 (2.67%)	
ypTNM					0.418
1	67 (66.34%)	33 (76.74%)	92 (78.63%)	54 (72.00%)	
2	2 (1.98%)	1 (2.33%)	7 (5.98%)	9 (12.00%)	
3	28 (27.72%)	9 (20.93%)	16 (13.68%)	12 (16.00%)	
4	4 (3.96%)	0 (0.00%)	2 (1.71%)	0 (0.00%)	
TRG					0.416
1	24 (23.76%)	6 (13.95%)	50 (42.74%)	19 (25.33%)	
2	26 (25.74%)	15 (34.88%)	21 (17.95%)	23 (30.67%)	
3	33 (32.67%)	16 (37.21%)	31 (26.50%)	19 (25.33%)	
4	14 (13.86%)	6 (13.95%)	13 (11.11%)	13 (17.33%)	
5	4 (3.96%)	0 (0.00%)	2 (1.71%)	1 (1.33%)	
Pleural effusion					1.0
No	78 (77.23%)	34 (79.07%)	87 (74.36%)	55 (73.33%)	
Yes	23 (22.77%)	9 (20.93%)	30 (25.64%)	20 (26.67%)	
Postoperativepneumonia					0.347
No	74 (73.27%)	27 (62.79%)	78 (66.67%)	51 (68.00%)	
Yes	27 (26.73%)	16 (37.21%)	39 (33.33%)	24 (32.00%)	
Recurrent laryngeal nerve paralysis					0.346
No	88 (87.13%)	40 (93.02%)	103 (88.03%)	67 (89.33%)	
Yes	13 (12.87%)	3 (6.98%)	14 (11.97%)	8 (10.67%)	
Anastomotic leak					1.0
No	95 (94.06%)	40 (93.02%)	109 (93.16%)	73 (97.33%)	
Yes	6 (5.94%)	3 (6.98%)	8 (6.84%)	2 (2.67%)	
Chylothorax					0.973
No	97 (96.04%)	42 (97.67%)	113 (96.58%)	70 (93.33%)	
Yes	4 (3.96%)	1 (2.33%)	4 (3.42%)	5 (6.67%)	
Postoperativedelirium					0.984
No	97 (96.04%)	42 (97.67%)	109 (93.16%)	73 (97.33%)	
Yes	4 (3.96%)	1 (2.33%)	8 (6.84%)	2 (2.67%)	
Length of hospital stay (days)	13.71 ± 2.43	13.68 ± 2.50	13.53 ± 2.51	13.57 ± 2.41	0.976

Note: Demographic, physiological, surgical, pathological, and inflammatory indices of all enrolled patients were summarized. No significant inter-cohort differences were observed (*p* > 0.05), indicating balanced group distribution for model development and validation

**Table 2:** Univariable and multivariable logistic regression analyses of clinical predictors for pathological complete response (pCR)

Feature name	Univariable OR (95% CI)		*P* value		Multivariable OR (95% CI)		*P* value	
Age	1.001	0.990–1.012	0.868					
Gender								
Female	Ref							
Male	1.079	0.934–1.246	0.384					
BMI	0.997	0.969–1.026	0.855					
FEV1/FVC%	0.999	0.993–1.005	0.764					
Hypertension								
No	Ref							
Yes	0.997	0.862–1.151	0.968					
Coronary Heart Disease								
No	Ref							
Yes	0.975	0.842–1.129		0.774				
Diabetes Mellitus								
No	Ref							
Yes	1.061	0.889–1.267		0.581				
Smoking								
No	Ref							
Yes	1.072	0.922–1.245		0.444				
Drinking								
No	Ref							
Yes	1.085	0.933–1.261		0.372				
LVEF	0.959	0.929–0.991		< 0.05	0.968	0.938–0.999		0.095
ASA PG								
I	Ref							
II	1.069	0.959–1.192		0.312				
III	1.114	0.973–1.274		0.188				
SII	1.000	1.000–1.000		0.25				
NLR	1.003	0.969–1.037		0.884				
PNI	0.996	0.987–1.005		0.436				
Ki67	0.988	0.942–1.038		0.694				
Tumor length	0.952	0.856–1.060		0.449				
Tumor location								
Upper	Ref							
Middle	0.992	0.896–1.102		0.870				
Low	1.017	0.919–1.125		0.781				
cT								
T1	Ref							
T2	1.462	1.238–1.724		< 0.001	1.392	1.175–1.651		< 0.001
T3	1.879	1.575–2.242		< 0.001	1.738	1.456–2.074		< 0.001
cN								
N0	Ref							
N1	0.971	0.846–1.115		0.690				
N2	1.009	0.815–1.081		0.459				
Chylothorax								
No	Ref							
Yes	0.755	0.518-1.100		0.218				
Anastomotic leak								
No	Ref							
Yes	0.895	0.655–1.224		0.558				
Pleural effusion								
No	Ref							
Yes	1.027	0.864–1.220		0.799				
Recurrent laryngeal nerve paralysis								
No	Ref							
Yes	1.104	0.892–1.366		0.442				
Postoperative pneumonia								
No	Ref							
Yes	1.106	0.942-1.300		0.299				
Postoperative delirium								
No	Ref							
Yes	1.645	1.135–2.382		< 0.05	1.458	1.011–2.102		0.09
Length of hospital stay	0.974	0.946–1.003		0.138				

Note: Odds ratios (OR), confidence intervals (CI), and p-values are shown for each variable. Statistically significant features (*p* < 0.05) were included in the clinical model or integrated model construction

### Radiomic feature analysis

**Fig. 3:**
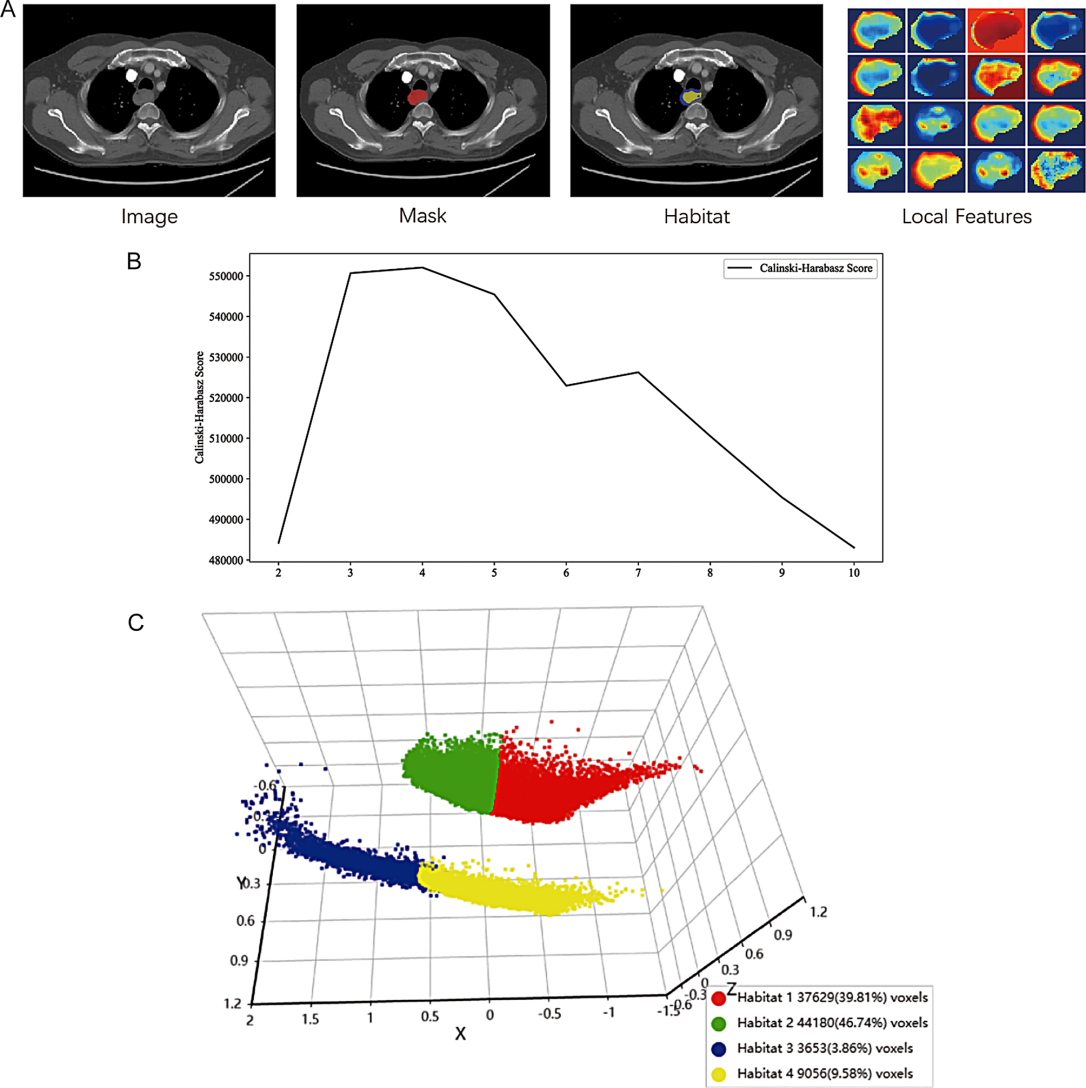
Visualization of the habitat-based subregional segmentation pipeline. **(A)** Representative axial CT slice illustrating the tumor region, corresponding manual segmentation mask, derived habitat subregions after unsupervised clustering, and voxel-level spatial feature maps derived from local texture descriptors. These local features capture spatial variations in tumor heterogeneity and form the basis for habitat delineation. **(B)** Calinski–Harabasz (CH) scores plotted across varying numbers of clusters (2–10), used to determine the optimal cluster count for habitat partitioning. A peak CH score was observed at four clusters, indicating optimal inter-cluster separability and compactness. **(C)** Three-dimensional visualization of the clustered feature space, demonstrating spatially distinct distributions of intratumoral subregions (habitats), with each cluster corresponding to a unique imaging phenotype

**Fig. 4:**
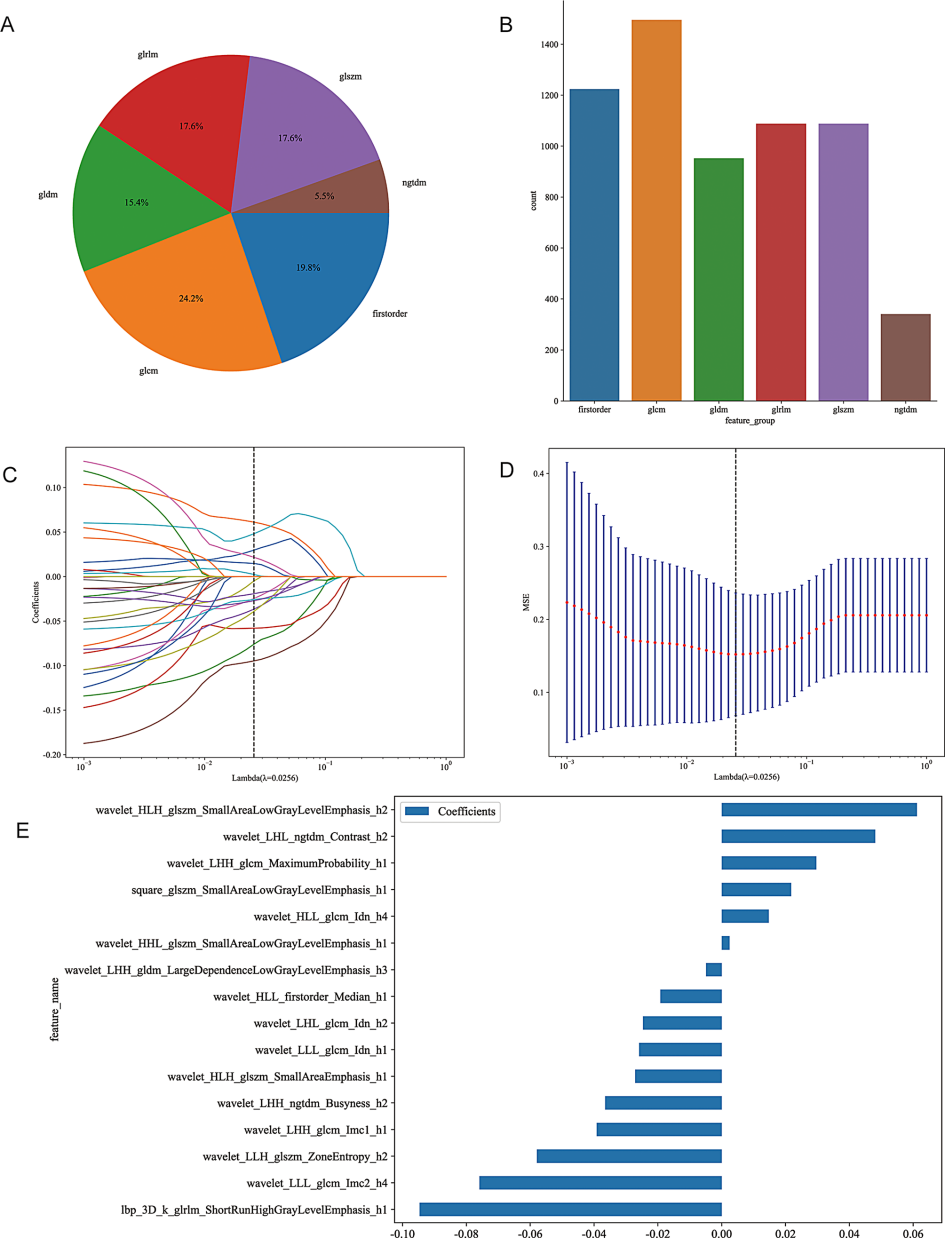
Characterization and selection of handcrafted radiomic features derived from tumor habitats. **(A)** Pie chart showing the proportional distribution of radiomic feature types extracted from all habitat subregions. Texture features derived from the gray-level co-occurrence matrix (GLCM) and gray-level run length matrix (GLRLM) constituted the majority, followed by first-order and shape descriptors. **(B)** Bar plot presenting the absolute count of features across different categories, indicating that GLCM and first-order features were the most abundant in the initial feature set. **(C)** LASSO coefficient trajectories for radiomic features plotted against the regularization parameter (lambda). Features with non-zero coefficients were retained, while others were shrunk to zero, aiding dimensionality reduction. **(D)** Ten-fold cross-validation curve for selecting the optimal lambda in LASSO regression, with the minimum mean squared error (MSE) and the corresponding confidence interval shown. The chosen lambda balances model complexity and prediction accuracy. **(E)** Bar graph visualizing the non-zero coefficients of the top features selected by LASSO regression. Feature names reflect both the image transformation (e.g., wavelet, square, lbp-3D) and the statistical property (e.g., entropy, emphasis), as well as the corresponding habitat index, illustrating their contribution to the final predictive model

The radiomic features were extracted from pre-treatment CT images for both traditional and habitat radiomics, as illustrated in Figs. 3 and 4. Traditional radiomic features included first-order statistics (e.g., mean, variance, skewness), texture features (e.g., GLCM, GLRLM, GLSZM), and shape features (e.g., volume, surface area, sphericity), all of which provided a global characterization of the tumor. On the other hand, habitat radiomic features were derived from tumor subregions using K-means clustering, allowing for more localized feature extraction from spatially distinct regions within the tumor. As shown in Fig. 3, segmenting the tumor into 4 distinct habitats enabled a more detailed analysis of tumor heterogeneity. Figure 4 demonstrates that habitat radiomic features, especially texture-related features like GLCM Contrast, GLRLM Long Run Emphasis, and GLSZM Small Area High Gray Level Emphasis, exhibited significantly higher importance scores in predicting pCR compared to traditional radiomic features. These features are key in assessing spatial variations in tumor heterogeneity, which directly contribute to treatment response prediction. The findings highlight that habitat radiomic features, particularly those reflecting intratumoral heterogeneity, provided superior predictive value for pCR compared to traditional features, underscoring the advantage of incorporating tumor microenvironmental heterogeneity into predictive models.

### Machine learning model performance

**Fig. 5:**
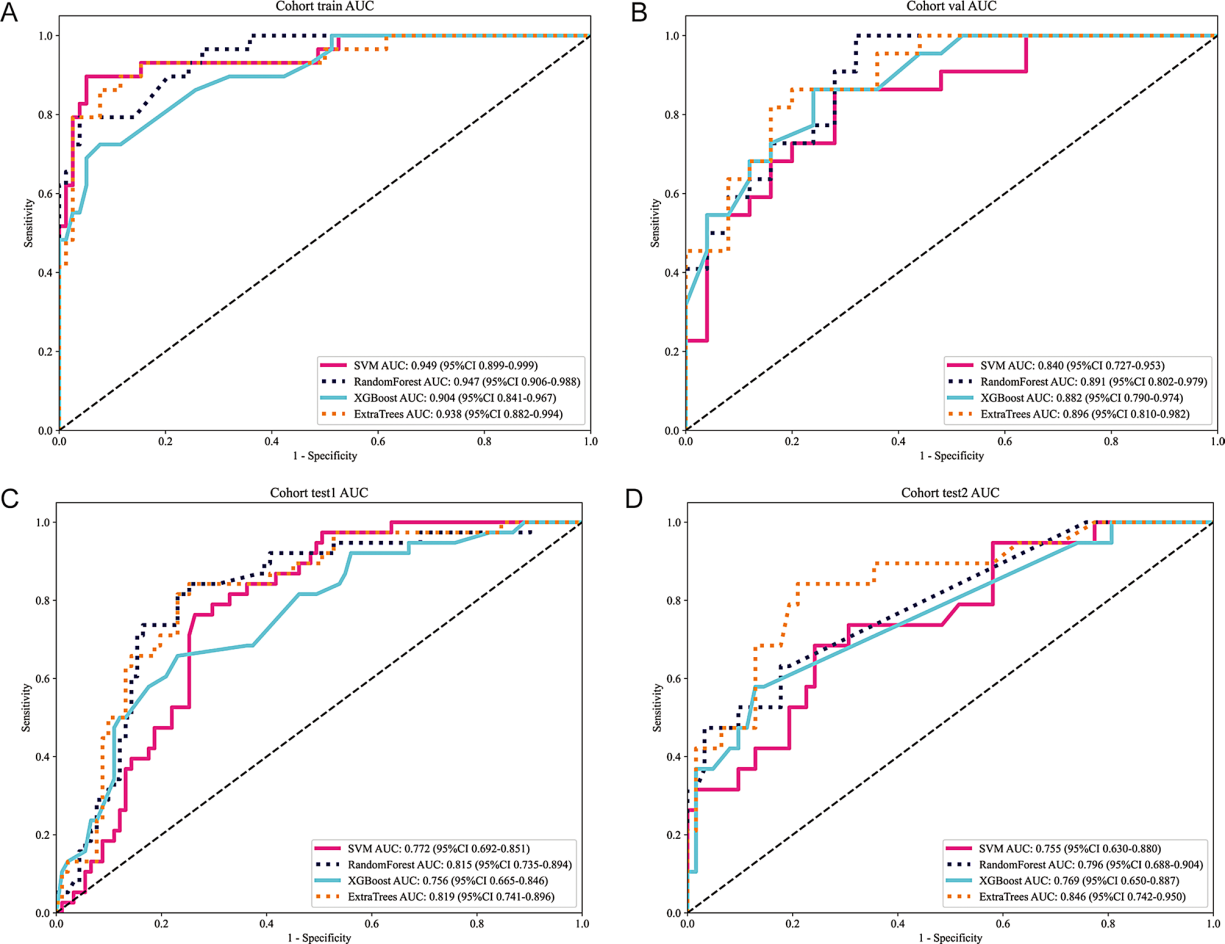
Receiver operating characteristic (ROC) curves of four machine learning models across different patient cohorts. **(A)** ROC curves for the training cohort demonstrate high discriminative performance across all models, with ExtraTrees achieving the area under the curve (AUC = 0.938). **(B)** In the internal validation cohort, ExtraTrees maintained strong generalization (AUC = 0.896), while the other models showed moderately lower performance, suggesting consistent predictive capacity across independent data. **(C)** ROC analysis in the first external test set reveals a comparable trend, with ExtraTrees (AUC = 0.819) outperforming other classifiers. **(D)** In the second external test set, ExtraTrees again yielded the highest AUC (0.846), demonstrating its robustness and stability across varying datasets. Across all cohorts, the habitat-derived radiomic signatures combined with advanced ensemble classifiers offered superior predictive accuracy for pCR following NACIT in ESCC

We evaluated the performance of several machine learning models trained on both traditional and habitat radiomic features. The models included Support Vector Machine (SVM), Random Forest, XGBoost, and ExtraTrees. Figure 5 illustrates the ROC curves for these models, showing their ability to distinguish between pCR and non-pCR cases across different cohorts. SVM achieved an AUC of 0.949 in the training cohort, 0.840 in the internal validation cohort, 0.772 in external test cohort 1, and 0.755 in external test cohort 2. Random Forest demonstrated strong performance, with AUCs of 0.947 in the training cohort, 0.891 in the internal validation cohort, 0.815 in external test cohort 1, and 0.796 in external test cohort 2. XGBoost yielded AUCs of 0.904 in the training cohort, 0.882 in the internal validation cohort, 0.756 in external test cohort 1, and 0.769 in external test cohort 2. ExtraTrees, another ensemble method, showed AUCs of 0.938 in the training cohort, 0.896 in the internal validation cohort, 0.819 in external test cohort 1, and 0.846 in external test cohort 2. Table 3 provides a summary of model performance across all cohorts, including sensitivity, specificity, PPV, and NPV. Among the models evaluated, ExtraTrees demonstrated the most robust performance across all cohorts, with the highest overall AUC and a strong balance between sensitivity and specificity. This model effectively captured both tumor heterogeneity and clinical data, offering superior predictive accuracy for pCR.

**Table 3:** Comparative performance of machine learning algorithms based on Habitat-Derived radiomic features in all cohorts

Model name	Accuracy	AUC	95% CI	Sensitivity	Specificity	PPV	NPV	Cohort
SVM	0.935	0.949	0.899–0.999	0.897	0.949	0.867	0.961	train
SVM	0.787	0.840	0.727–0.953	0.864	0.720	0.731	0.857	val
SVM	0.744	0.772	0.692–0.851	0.763	0.736	0.547	0.882	test1
SVM	0.741	0.755	0.630–0.880	0.684	0.758	0.464	0.887	test2
RandomForest	0.916	0.947	0.906–0.988	0.793	0.962	0.885	0.926	train
RandomForest	0.830	0.891	0.802–0.979	1.000	0.680	0.733	1.000	val
RandomForest	0.775	0.815	0.735–0.894	0.842	0.747	0.582	0.919	test1
RandomForest	0.778	0.796	0.688–0.904	0.632	0.823	0.522	0.879	test2
XGBoost	0.869	0.904	0.841–0.967	0.724	0.923	0.778	0.900	train
XGBoost	0.809	0.882	0.790–0.974	0.864	0.760	0.760	0.864	val
XGBoost	0.736	0.756	0.665–0.846	0.658	0.769	0.543	0.843	test1
XGBoost	0.802	0.769	0.650–0.887	0.579	0.871	0.579	0.871	test2
ExtraTrees	0.907	0.938	0.882–0.994	0.862	0.923	0.806	0.947	train
ExtraTrees	0.830	0.896	0.810–0.982	0.864	0.800	0.792	0.870	val
ExtraTrees	0.775	0.819	0.741–0.896	0.842	0.747	0.582	0.919	test1
ExtraTrees	0.802	0.846	0.742–0.950	0.842	0.790	0.552	0.942	test2

Note: Four classifiers (SVM, Random Forest, XGBoost, and ExtraTrees) were evaluated for pCR prediction. Metrics included AUC, accuracy, sensitivity, specificity, PPV, and NPV across training, validation, and external test sets

### Comparison of habitat and radiomic models

**Fig. 6:**
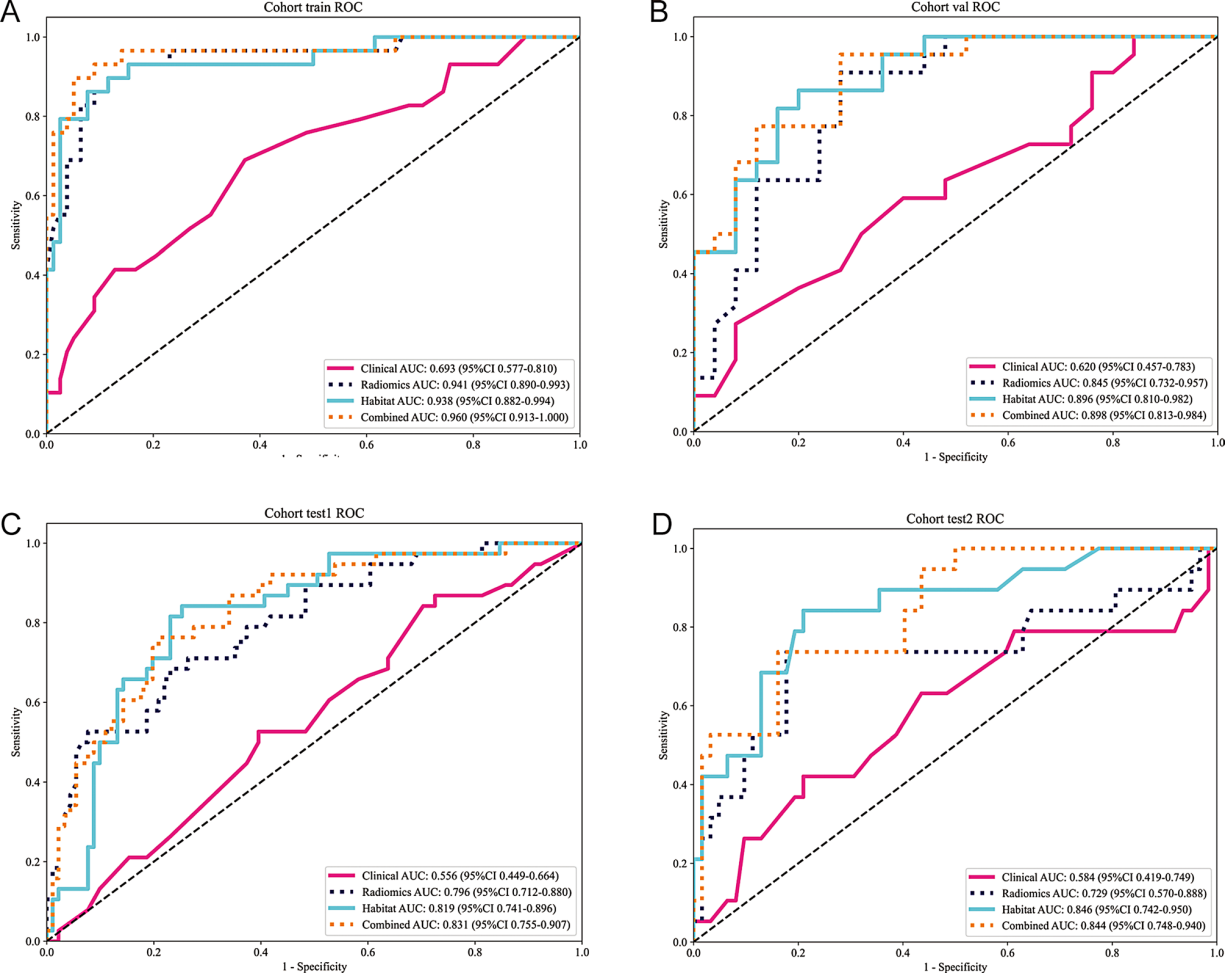
ROC curve comparison of different predictive signatures across all patient cohorts. **(A–D)** Receiver operating characteristic (ROC) curves of the Clinical, Radiomics, Habitat, and Combined models in the training **(A)**, internal validation **(B)**, and external test cohorts (**C**: test1; **D**: test2). In each cohort, the Habitat and Combined models achieved higher AUCs than the Clinical and conventional Radiomics models, indicating the added predictive value of spatially informed intratumoral subregional features

**Fig. 7:**
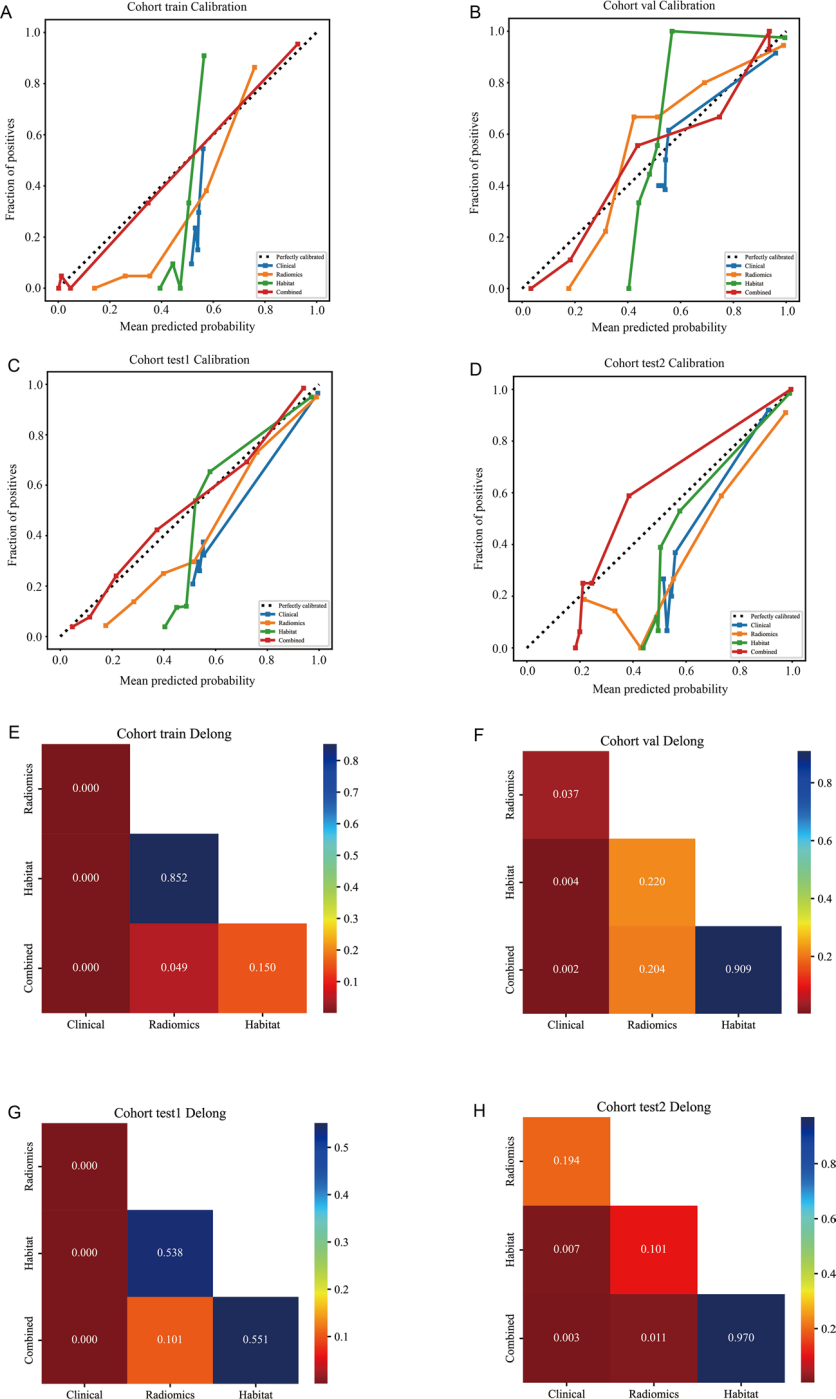
Calibration and pairwise AUC comparisons of predictive models. **(A–D)** Calibration curves for the Clinical, Radiomics, Habitat, and Combined models in the training **(A)**, internal validation **(B)**, and external test cohorts (**C**: test1; **D**: test2). The Combined and Habitat models showed closer alignment with the diagonal reference line, suggesting better agreement between predicted and observed probabilities. **(E–H)** Heatmaps of pairwise DeLong tests comparing AUCs among the four models in the same cohorts (**E**: train; **F**: internal validation; **G**: test1; **H**: test2). Lower p-values indicate significant performance differences. Across cohorts, the Habitat and Combined models generally outperformed the Clinical model, whereas differences between the Habitat and Radiomics models were often not statistically significant, and the Combined model provided only modest incremental gains over the Habitat model

A comparative analysis of habitat radiomics and traditional radiomics was conducted, with the results presented in Fig. 6; Table 4. The habitat radiomics model consistently outperformed the traditional radiomics model in terms of AUC, sensitivity, and specificity. In the training cohort, the habitat radiomics model achieved an AUC of 0.938, while the traditional radiomics model had an AUC of 0.941. In the internal validation cohort, the habitat model had an AUC of 0.896, compared to 0.845 for the traditional radiomics model. Similarly, in external test cohort 1, the habitat radiomics model achieved an AUC of 0.819, outperforming the traditional radiomics model (AUC = 0.796). In external test cohort 2, the habitat radiomics model achieved an AUC of 0.846, substantially higher than that of the traditional radiomics model (AUC = 0.729). This performance gap was statistically significant (*p* < 0.05, DeLong test), highlighting the superior ability of habitat radiomics to capture the tumor’s spatial heterogeneity, which is crucial for predicting immunotherapy response in ESCC, as shown in Fig. 7. The combined model, which integrates both habitat and traditional radiomics features, showed the best performance across all cohorts. As shown in Fig. 6, the combined model achieved the highest performance, with an AUC of 0.960 in the training cohort, 0.898 in the internal validation cohort, 0.831 in external test cohort 1, and 0.844 in external test cohort 2. This model significantly improved the predictive accuracy for pCR, with notable improvements in both sensitivity (0.91 vs. 0.89) and specificity (0.93 vs. 0.91) when compared to the individual models.

**Table 4:** Predictive performance comparison of Clinical, Radiomics, Habitat-Based, and combined signatures for pCR prediction

Signature	Accuracy	AUC	95% CI	Sensitivity	Specificity	PPV	NPV	Cohort
Clinical	0.645	0.693	0.5772–0.8096	0.690	0.628	0.408	0.845	train
Radiomics	0.888	0.941	0.8895–0.9933	0.897	0.885	0.743	0.958	train
Habitat	0.907	0.938	0.8819–0.9944	0.862	0.923	0.806	0.947	train
Combined	0.935	0.960	0.9133–1.0000	0.897	0.949	0.867	0.961	train
Clinical	0.468	0.620	0.4570–0.7830	1.000	0.000	0.468	0.000	val
Radiomics	0.723	0.845	0.7319–0.9572	0.545	0.880	0.800	0.687	val
Habitat	0.766	0.896	0.8103–0.9824	0.636	0.880	0.824	0.733	val
Combined	0.809	0.898	0.8126–0.9837	0.773	0.840	0.810	0.808	val
Clinical	0.295	0.556	0.4488–0.6637	1.000	0.000	0.295	0.000	test1
Radiomics	0.736	0.796	0.7115–0.8799	0.553	0.813	0.553	0.813	test1
Habitat	0.760	0.819	0.7413–0.8960	0.737	0.769	0.571	0.875	test1
Combined	0.783	0.831	0.7552–0.9065	0.500	0.901	0.679	0.812	test1
Clinical	0.235	0.584	0.4193–0.7488	1.000	0.000	0.235	0.000	test2
Radiomics	0.716	0.729	0.5701–0.8875	0.737	0.710	0.437	0.898	test2
Habitat	0.790	0.846	0.7418–0.9500	0.789	0.790	0.536	0.925	test2
Combined	0.778	0.844	0.7478–0.9398	0.105	0.984	0.667	0.782	test2

Note: The Combined model integrating habitat radiomics and clinical features achieved the highest overall performance in training and validation cohorts. The Habitat model demonstrated superior generalizability in external test sets

### Clinical utility and decision support

**Fig. 8:**
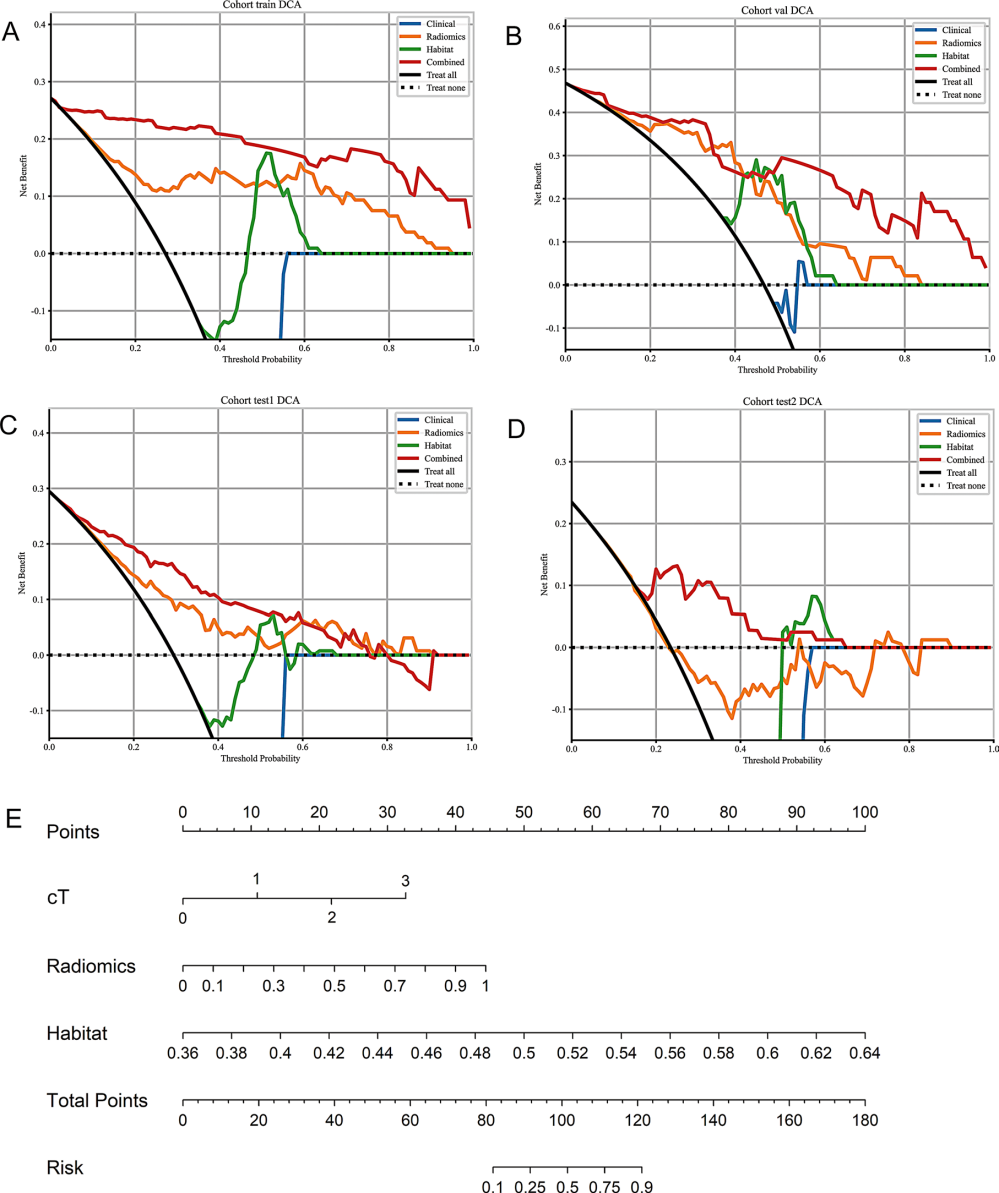
Clinical utility assessment and nomogram based on the Combined model. **(A–D)** Decision curve analysis (DCA) of the Clinical, Radiomics, Habitat, and Combined models in the training **(A)**, internal validation **(B)**, and external test cohorts (**C**: test1; **D**: test2). Across a broad range of threshold probabilities, the Combined model yielded the highest net benefit, with the Habitat model also consistently outperforming the Clinical and Radiomics models alone. **(E)** Nomogram derived from the Combined model, integrating cT stage, Radiomics score, and Habitat score to estimate the individualized probability of achieving pCR following NACIT. Each variable contributes a point value on the upper scale; the summed points correspond to the predicted risk on the bottom scale, providing a practical tool for personalized decision support

The clinical utility of the models was further assessed using DCA, as presented in Fig. 7. The combined model demonstrated the highest net benefit across all threshold probabilities, indicating its potential to provide valuable guidance in clinical decision-making. The habitat radiomics model also showed a favorable net benefit, outperforming both the clinical and traditional radiomics models at a wide range of threshold probabilities. Furthermore, the nomogram derived from the combined model, as shown in Fig. 8, integrates clinical features (e.g., cT stage) with radiomic scores to predict individualized probabilities of pCR. This nomogram could serve as a clinical decision support tool, helping clinicians make more informed treatment decisions based on personalized risk assessments. Supplementary Material provides additional validation of the model’s clinical applicability and potential for improving patient outcomes. Supplementary Table 1 presents the clinical decision thresholds and associated net benefits, reinforcing the practical significance of the combined model in real-world clinical settings.

## Discussion

This study represents a significant advancement in the prediction of pCR for esophageal squamous cell carcinoma (ESCC) patients undergoing neoadjuvant immunotherapy combined with chemotherapy. We developed and validated machine learning models that integrate both habitat and traditional radiomics features to predict pCR. Our findings demonstrate that habitat radiomics, which captures tumor heterogeneity at a spatial level, outperforms traditional radiomics in predicting pCR. The combined model, which integrates both habitat and traditional radiomics features, provided the best performance with an AUC of 0.960 in the training cohort, with strong performance retained across external cohorts (0.819–0.846). The use of a nomogram developed from these models offers a clinically valuable tool that combines radiomic and clinical data to predict pCR in ESCC patients. Our study is consistent with existing literature on the predictive capabilities of radiomics in oncology but further extends these concepts by introducing habitat radiomics, a method that better reflects the spatial heterogeneity of the tumor microenvironment, an important factor in immunotherapy response [8, 21, 22].

One of the key findings of this study is the superior predictive performance of habitat radiomics over conventional radiomics. By segmenting the tumor into distinct subregions (“habitats”), this method captures spatially resolved phenotypic variations that likely reflect underlying biological and microenvironmental heterogeneity, in line with contemporary standards for rigorous image biomarker extraction and reporting [23]. Recent multicenter investigations in ESCC have demonstrated that spatially informed approaches, such as habitat- or voxel-level radiomics, are closely associated with immunotherapy efficacy and exhibit reproducible predictive value in neoadjuvant immunotherapy cohorts [24–26]. Furthermore, a growing body of evidence indicates that the spatial distribution of texture features—rather than global, whole-tumor averages—correlates strongly with response to immunotherapy or chemoradiotherapy [27]. In our cohort, texture heterogeneity–related descriptors, including gray-level co-occurrence matrix (GLCM) contrast and gray-level run-length matrix (GLRLM) long-run emphasis, demonstrated substantial discriminative power, consistent with prior studies showing that incorporating texture- and peritumor-derived information can enhance predictive accuracy [9, 28].

Although our pipeline delineates intratumoral subregions based on voxel-level phenotypes, the present study did not perform direct radiology–pathology co-localization. To enhance interpretability, future work will co-register habitat maps with surgical specimens for region-matched histopathology and multi-omic profiling to test whether imaging-defined habitats correspond to specific microenvironmental states. Prior studies suggest that metabolic or MRI-based habitat phenotypes can align with treatment-response biology, supporting the feasibility of such validation [29]. Collectively, these steps will help establish links between habitat labels and underlying pathobiology, thereby strengthening clinical translation.

Beyond prediction, several top-ranked habitat-derived texture descriptors in our study (e.g., GLCM contrast, GLRLM long-run emphasis, GLSZM small-area high gray-level emphasis) plausibly map to distinct microenvironmental states. Habitats with higher contrast/entropy likely arise at viable–necrotic interfaces or hypoxic/peritumoral regions, where sharp intensity transitions reflect edema, necrosis, or active immune–tumor interaction; such regions may be enriched for CD8⁺ T cells, tumor-associated macrophages, and/or PD-L1 expression [30]. In contrast, habitats with higher long-run emphasis (extended homogeneous runs) are consistent with fibrotic/keratinized stroma and lower immune infiltration, akin to an “immune-desert” phenotype [31]. These interpretations are hypothesis-generating in this retrospective design. To substantiate them prospectively, we will perform region-matched immunohistochemistry (CD8, PD-L1, CD68/CD163, Ki-67, CD31, α-SMA) and collagen staining (e.g., Masson’s trichrome/picrosirius red), together with spatial transcriptomic profiling of hypoxia, proliferation, and stromal signatures [32, 33].

While our study provides valuable insights, several statistical limitations should be acknowledged. First, we calculated the 95% confidence intervals (CIs) for the Area Under the Curve (AUC) using DeLong tests based on random splits and fixed external test cohorts. However, since we did not perform repeated cross-validation for all performance metrics, CIs for sensitivity and specificity could not be calculated. Additionally, DeLong tests were performed without correction for multiple comparisons, which could potentially introduce bias when comparing the models’ performance across multiple cohorts. Moreover, the internal validation cohort contained a relatively small sample size (*n* = 43). This small sample size may limit the robustness of the reported AUC values and affect the generalizability of the findings. Future work should focus on larger datasets and more rigorous cross-validation techniques to confirm the stability and reproducibility of the results across different patient populations. Formal test–retest reliability of handcrafted radiomic features (e.g., repeat-scan ICC/CCC) was not assessed in this retrospective, multicenter dataset due to the lack of repeat acquisitions. As a pragmatic proxy, we monitored selection consistency across inner cross-validation folds during LASSO-based feature selection. In prospective work, we plan to incorporate test–retest or phantom imaging to quantify stability more rigorously and to integrate stability-selection procedures within the modeling pipeline.

Looking ahead, habitat radiomics has the potential to be integrated into clinical workflows and prospective trials to further refine treatment prediction [34]. Future studies should explore how these models can be utilized for real-time assessment of treatment response, providing dynamic feedback that could guide personalized therapy adjustments [35]. Given the ability of habitat radiomics to capture spatial heterogeneity at a more granular level, it could be especially beneficial in identifying responders to immunotherapy early on, thus improving patient outcomes. In terms of prospective trials, it would be useful to test the combined model in multi-center, randomized controlled trials to assess its generalizability and clinical utility across different patient populations [36]. Additionally, voxel-based radiomics focuses on extracting features from each individual voxel (3D pixel) of the tumor, providing a highly detailed analysis of the spatial characteristics of the tumor [37]. However, this approach may be limited by the complexity and volume of data, often requiring large computational resources. On the other hand, delta-radiomics analyzes the changes in radiomic features over time (e.g., before and after treatment), providing valuable insight into treatment response dynamics [38]. Habitat radiomics, as used in our study, segments the tumor into spatially distinct regions or “habitats” based on voxel-level features, focusing on capturing the intratumoral heterogeneity that might be missed by global features. By capturing more localized variations, habitat radiomics offers a more nuanced understanding of the tumor microenvironment and may be particularly beneficial in predicting the efficacy of treatments such as immunotherapy [39]. In the present study, we employed a conventional unsupervised clustering approach for habitat partitioning (k-means with data-driven selection of cluster number) to ensure transparency, reproducibility, and computational tractability across multicenter cohorts. We recognize that more advanced spatial modeling—including graph-based learning on super-voxel graphs, topological data analysis (e.g., persistent homology), and spatial mixture/Markov random-field formulations—may better capture non-linear spatial dependencies and topological structure. A comprehensive head-to-head evaluation of these methods is planned for future work, where we will benchmark predictive performance (AUC with confidence intervals, calibration, and decision-curve net benefit), spatial stability under resampling, computational cost, and clinical interpretability within a unified pipeline.

Beyond the spatial partitioning addressed in this work, modeling non-linear, high-dimensional medical imaging data remains challenging due to manifold non-linearity, the curse of dimensionality, susceptibility to spurious correlations, and incomplete data (e.g., missing slices/time points or heterogeneous acquisition) [40, 41]. While our current pipeline mitigates some of these issues via strict harmonization and nested validation, future extensions will consider deep non-linear factorization and attention mechanisms tailored to spatio-temporal heterogeneity [42]. In particular, time–frequency / spatial–frequency representations combined with attention—such as Fourier attention and wavelet attention—offer theoretically grounded means to capture multi-scale structure and long-range interactions while remaining robust to incomplete observations [43]. Concretely for habitat radiomics, spectral tokens could be derived from voxel-level or habitat-level feature maps (e.g., applying FFT/DWT along spatial axes and, when available, along longitudinal scans in delta-radiomics), with attention used to weight salient frequency bands or spatial scales [44]. Such architectures can enhance sensitivity to fine-grained patterns at viable–necrotic interfaces and hypoxic rims, suppress acquisition-induced noise, and provide interpretable frequency-scale attributions that complement habitat labels [45]. We emphasize that these approaches were not applied in the present study; rather, they constitute a prospective roadmap to augment the current habitat framework, to be benchmarked alongside graph-/topology-aware models within unified, externally validated pipelines.

We evaluated multiple machine learning algorithms, including Support Vector Machines (SVM), Random Forest, XGBoost, and ExtraTrees. Evidence from ESCC radiomics studies indicates that ensemble and gradient-boosting methods generally achieve superior generalizability for pCR prediction, while SVM often demonstrates an advantage in sensitivity [21, 46]. In multicenter investigations and models integrating deep feature representations, combining clinical or hematologic parameters with imaging-derived features has been shown to further enhance both discriminative performance and model robustness [25]. In our analysis, the ExtraTrees algorithm achieved the highest AUC, sensitivity, and specificity, aligning with recent reports comparing the performance of diverse machine learning approaches in similar oncologic prediction tasks [47].

With the expanding role of immunotherapy in the neoadjuvant treatment of esophageal squamous cell carcinoma, accurately identifying patients who are likely to achieve pCRis critical for optimizing therapeutic strategies. Systematic reviews and randomized or prospective trials have provided robust evidence supporting the clinical efficacy and safety of NACIT in resectable ESCC [48]. Integrating radiomics—particularly habitat- or voxel-level approaches—with key clinical variables into visual decision-support tools such as nomograms holds considerable promise for guiding individualized perioperative management [49]. Moreover, multimodal integration of PET/CT and CT-derived radiomic features with clinical data has demonstrated added predictive value for pCR, representing a practical paradigm for image–clinical fusion in precision oncology [50].

Conventional biomarkers and clinical staging offer limited discriminatory power in predicting immunotherapy response, whereas subregional or temporal CT/MR-based radiomics provides a more comprehensive depiction of the tumor microenvironment and intratumoral heterogeneity, thereby enhancing the accuracy of pCR prediction and reducing the risk of unnecessary treatment [21, 51].

While our study provides valuable insights, there are several limitations. First, although the study utilized a multicenter cohort, the patient sample was limited to a specific region, which could affect the generalizability of the results to other populations. Future studies should involve a more diverse patient population to confirm the robustness of the models across different genetic backgrounds and geographic regions. Second, while intraclass correlation coefficients (ICC) were used to assess segmentation consistency, some variability in tumor segmentation is inevitable. This is a common limitation in radiomics studies and can affect the reproducibility of results. Future studies should consider incorporating automated segmentation techniques to minimize this variability. Lastly, although our study incorporated important clinical variables, other biomarkers such as genetic mutations or immune profiling may improve prediction accuracy [52]. Moreover, we acknowledge that the present study lacked direct radiology–pathology co-localization and molecular validation of imaging-defined habitats; this will be prioritized in prospective studies to biologically substantiate habitat labels and strengthen clinical interpretability.

The integration of such biomarkers could provide a more comprehensive understanding of tumor biology, particularly in the context of immunotherapy response [53]. To further validate the models developed in this study, prospective clinical trials involving a more diverse and larger patient cohort are necessary. A prospective study would allow for the real-world validation of our nomogram and provide further evidence of its clinical utility.

To strengthen the evidence base for our models, large-scale, multicenter prospective trials involving more diverse patient populations are essential. Such studies would enable real-world validation of the nomogram and provide robust confirmation of its clinical utility, building on methodological precedents set by recent high-quality NICRT/NICT and chemoradiotherapy trials [54]. Integrating genomic and proteomic biomarkers—such as minimal residual disease (MRD) profiles and immune repertoire signatures—into radiomics-based frameworks may yield deeper mechanistic insights into immunotherapy response while enhancing predictive accuracy [55, 56]. In parallel, the implementation of fully automated, deep learning–driven segmentation and feature extraction pipelines, which have already demonstrated predictive value in ESCC patients undergoing nCRT, should be standardized and validated in immunotherapy-treated cohorts [57]. Furthermore, the underlying principles of habitat-, voxel-level-, and delta-radiomics have shown promise for predicting pCR or major pathological response (MPR) in other immunotherapy-sensitive malignancies, including non–small cell lung cancer (NSCLC), supporting the broader applicability of this approach across cancer types [58].

## Conclusion

This study highlights the promising role of habitat radiomics in improving the prediction of pathological complete response in ESCC patients receiving neoadjuvant immunotherapy and chemotherapy. By incorporating tumor heterogeneity into predictive models, we provide a more accurate and personalized approach to treatment prediction. The combined model, along with the developed nomogram, offers a valuable tool for clinicians to make more informed decisions and optimize treatment strategies for ESCC patients. Future validation in diverse cohorts, along with the integration of additional biomarkers, will further enhance the model’s clinical utility and expand its applicability to other cancers and therapies.

## Supplementary Information

Below is the link to the electronic supplementary material.

Supplementary Material 1

## Data Availability

The corresponding author can be contacted to request access to the datasets used and/or analyzed during the current study. Please reach out to the corresponding author for further information regarding the availability of the datasets.
