# Supplementary material for: Integration of habitat radiomics and traditional radiomic features for predicting pathological complete response in esophageal squamous cell carcinoma following neoadjuvant immunotherapy and chemotherapy: a multicenter comparative study
Source: J Transl Med. 2026 Jan 13;24:231. doi: 10.1186/s12967-025-07522-y (PMC12903750; doi:10.1186/s12967-025-07522-y)
Supplement: Supplementary file 1 — Supplementary Material 1 [file 12967_2025_7522_MOESM1_ESM.docx]

## Supplementary

### 1A. Habitat Generation Process

Our methodology for delineating tumor habitat regions was multifaceted and involved several complex steps:

1. **Comprehensive Radiomic Feature Extraction:** This process involved extracting detailed local features from each voxel in the dataset using a $5\times5$ moving window. These features encompass a variety of measurements and attributes, including intensity, texture, and other statistical properties, which are crucial for understanding the intricate details of the dataset. Such detailed insights enable more precise modeling and analysis.

- In this study, 19 radiomic features were extracted from each voxel, offering a multidimensional characterization of each subregion. These features included a range of shape descriptors, textural features, and first-order statistical attributes. The specific features extracted were: firstorder_Entropy, firstorder_MeanAbsoluteDeviation, firstorder_Median, glcm_DifferenceAverage, glcm_DifferenceEntropy, glcm_DifferenceVariance, glcm_Imc1, glcm_Imc2, glcm_InverseVariance, glcm_JointEnergy, glcm_JointEntropy, glcm_SumEntropy, glrlm_LongRunEmphasis, glrlm_RunEntropy, glrlm_RunVariance, glszm_SizeZoneNonUniformityNormalized, glszm_SmallAreaHighGrayLevelEmphasis, ngtdm_Contrast, and ngtdm_Strength.

1. **In-depth Clustering Analysis:** The K-means algorithm was utilized to analyze the multidimensional feature space derived from the radiomic features. The algorithm was applied with varying numbers of cluster centers, ranging from 3 to 9, to categorize distinct habitat regions within the tumor. The performance of the clustering was evaluated using the Calinski-Harabasz score, ensuring the selection of the most statistically significant clustering arrangement.

- The K-means algorithm functions by partitioning data into K distinct clusters. It iteratively updates the centroids of these clusters to minimize the sum of squares within each cluster. The central component of the K-means algorithm is the objective function, which is optimized to achieve effective clustering.

$$J=\sum_{i=1}^{N} \sum_{k=1}^{K} w_{ik}\times\parallel x_{i}-\mu_{k}\parallel^{2}$$

- - $J$ is the objective function.
  - $N$ is the number of data points.
  - $K$ is the number of clusters.
  - $w_{ik}$ is a binary indicator (1 if data point $i$ is in cluster $k$, 0 otherwise).
  - $x_{i}$ is the ith data point.
  - $\mu_{k}$ is the centroid of cluster $k$.
  - $\parallel x_{i}-\mu_{k}\parallel^{2}$ is the squared Euclidean distance between data point $i$ and centroid $k$.

1. **Habitat Region Synthesis:** Following the clustering analysis, subregions with identical cluster IDs were amalgamated. This synthesis resulted in the formation of comprehensive habitat regions, each representing a unique microenvironmental characteristic within the tumor.

### 2A. Details of Clinical Model


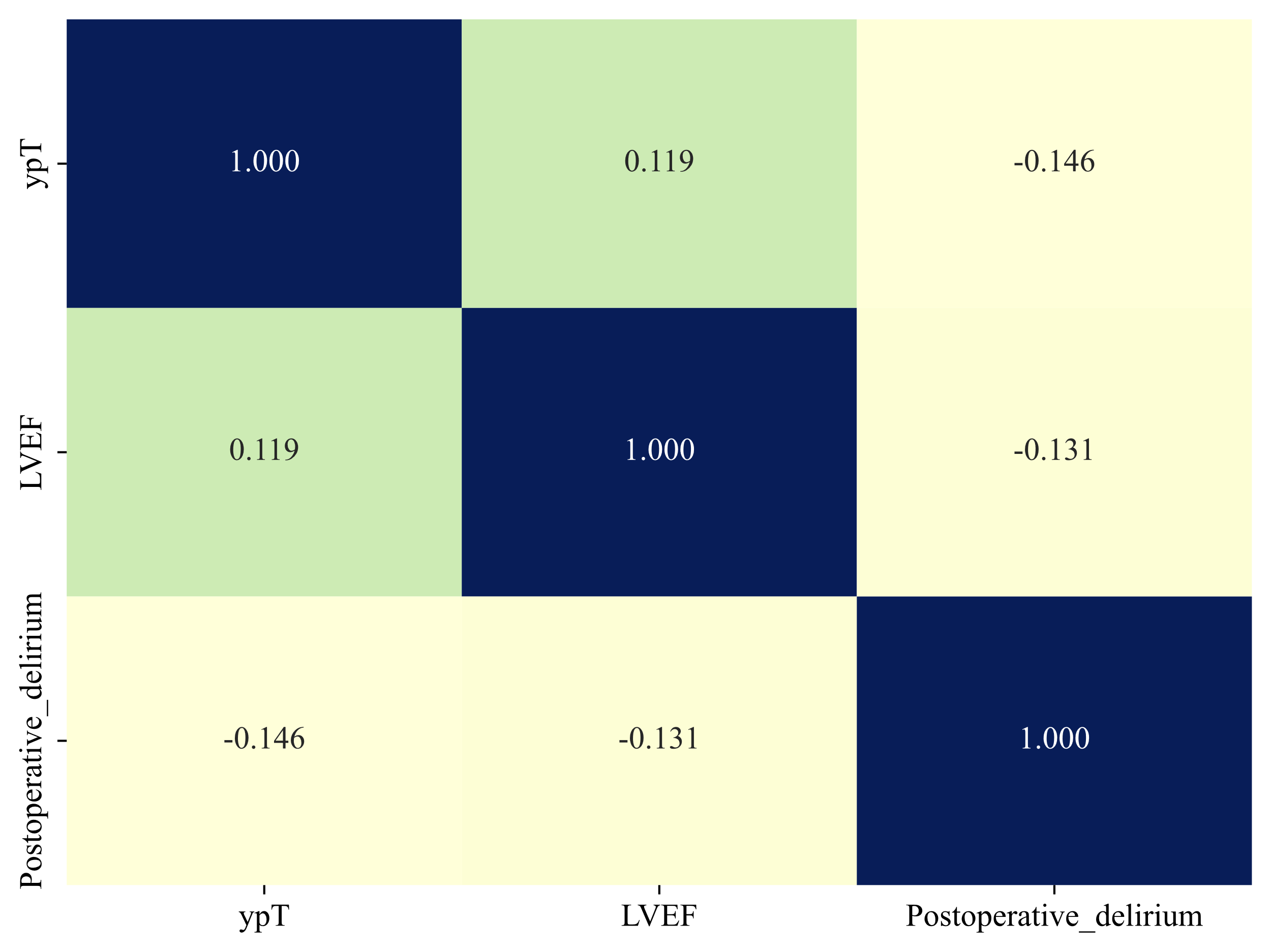


Fig. Correlation of Clinical Features

| model_name | Accuracy | AUC | 95% CI | Sensitivity | Specificity | PPV | NPV | Cohort |
| --- | --- | --- | --- | --- | --- | --- | --- | --- |
| SVM | 0.458 | 0.691 | 0.575 - 0.807 | 0.828 | 0.321 | 0.312 | 0.833 | train |
| SVM | 0.489 | 0.620 | 0.457 - 0.783 | 0.727 | 0.280 | 0.471 | 0.538 | val |
| SVM | 0.450 | 0.556 | 0.448 - 0.663 | 0.868 | 0.275 | 0.333 | 0.833 | test1 |
| SVM | 0.259 | 0.584 | 0.419 - 0.749 | 0.789 | 0.097 | 0.211 | 0.600 | test2 |
| RandomForest | 0.654 | 0.717 | 0.607 - 0.827 | 0.759 | 0.615 | 0.423 | 0.873 | train |
| RandomForest | 0.511 | 0.553 | 0.385 - 0.721 | 0.818 | 0.240 | 0.486 | 0.600 | val |
| RandomForest | 0.426 | 0.511 | 0.397 - 0.625 | 0.684 | 0.319 | 0.295 | 0.707 | test1 |
| RandomForest | 0.420 | 0.611 | 0.460 - 0.761 | 0.789 | 0.306 | 0.259 | 0.826 | test2 |
| ExtraTrees | 0.607 | 0.667 | 0.551 - 0.783 | 0.655 | 0.590 | 0.373 | 0.821 | train |
| ExtraTrees | 0.596 | 0.564 | 0.400 - 0.728 | 0.500 | 0.680 | 0.579 | 0.607 | val |
| ExtraTrees | 0.504 | 0.562 | 0.457 - 0.668 | 0.553 | 0.484 | 0.309 | 0.721 | test1 |
| ExtraTrees | 0.617 | 0.612 | 0.448 - 0.777 | 0.526 | 0.645 | 0.312 | 0.816 | test2 |
| XGBoost | 0.645 | 0.711 | 0.601 - 0.821 | 0.690 | 0.628 | 0.408 | 0.845 | train |
| XGBoost | 0.489 | 0.538 | 0.374 - 0.702 | 0.727 | 0.280 | 0.471 | 0.538 | val |
| XGBoost | 0.488 | 0.525 | 0.412 - 0.638 | 0.658 | 0.418 | 0.321 | 0.745 | test1 |
| XGBoost | 0.420 | 0.601 | 0.454 - 0.747 | 0.789 | 0.306 | 0.259 | 0.826 | test2 |


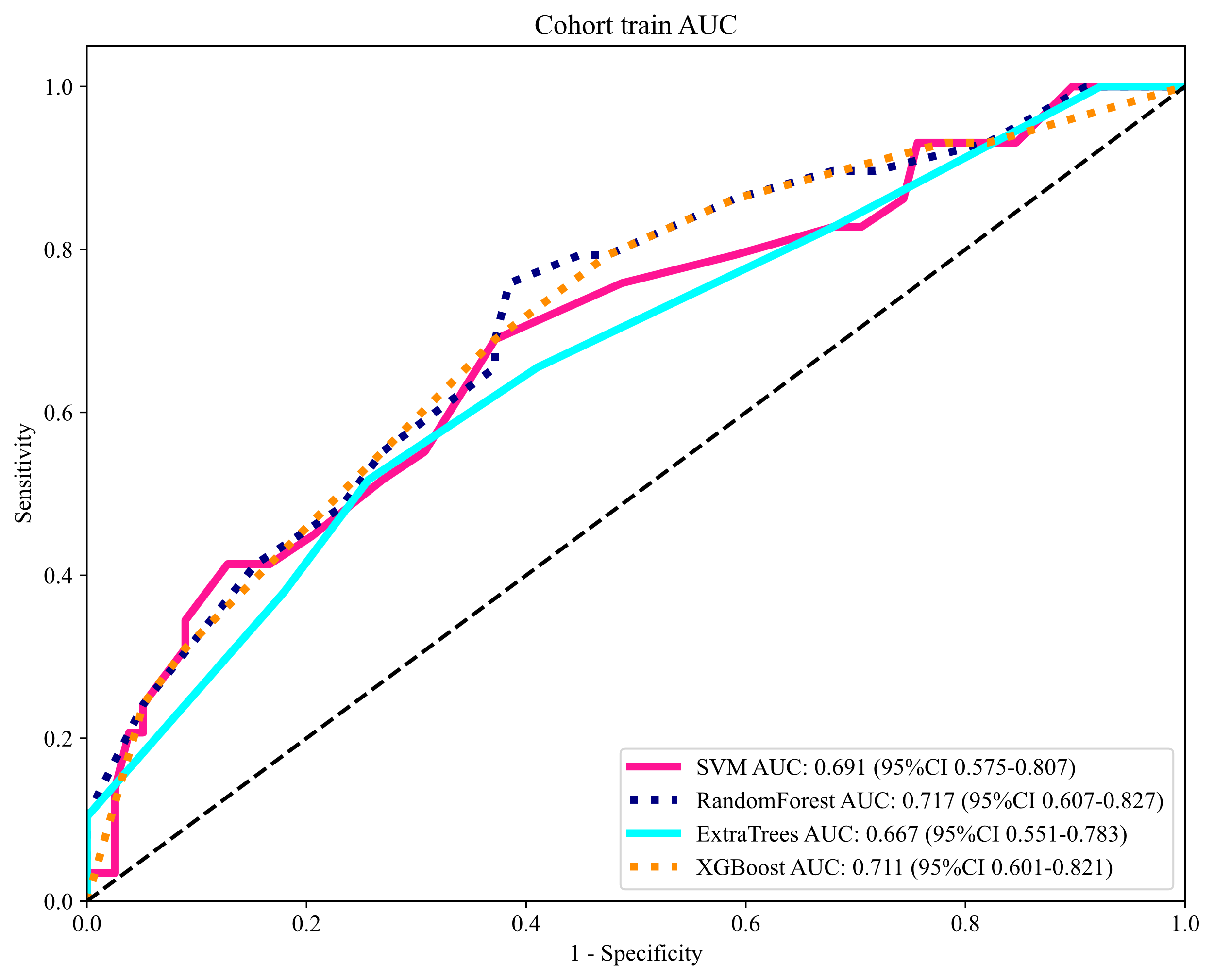

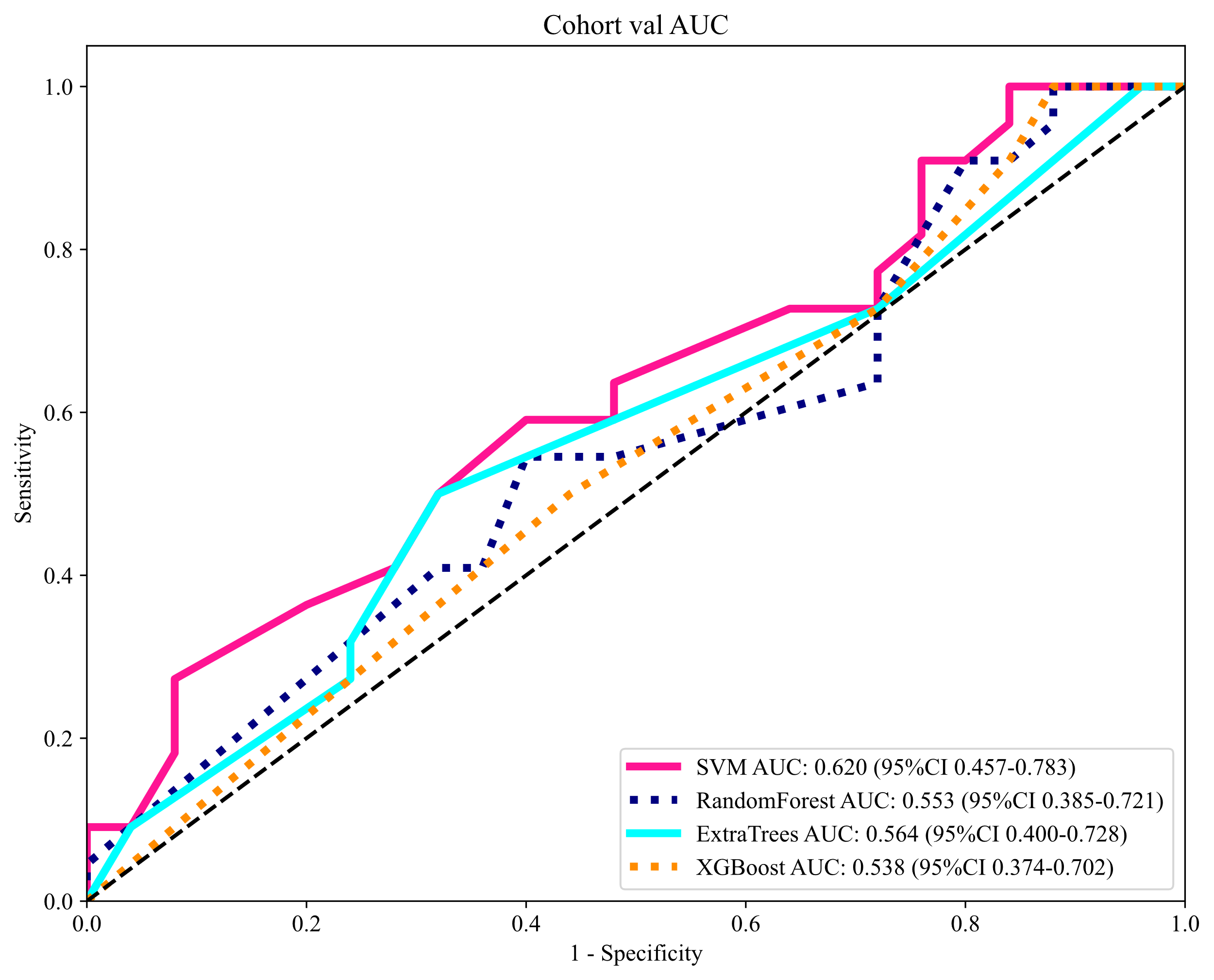

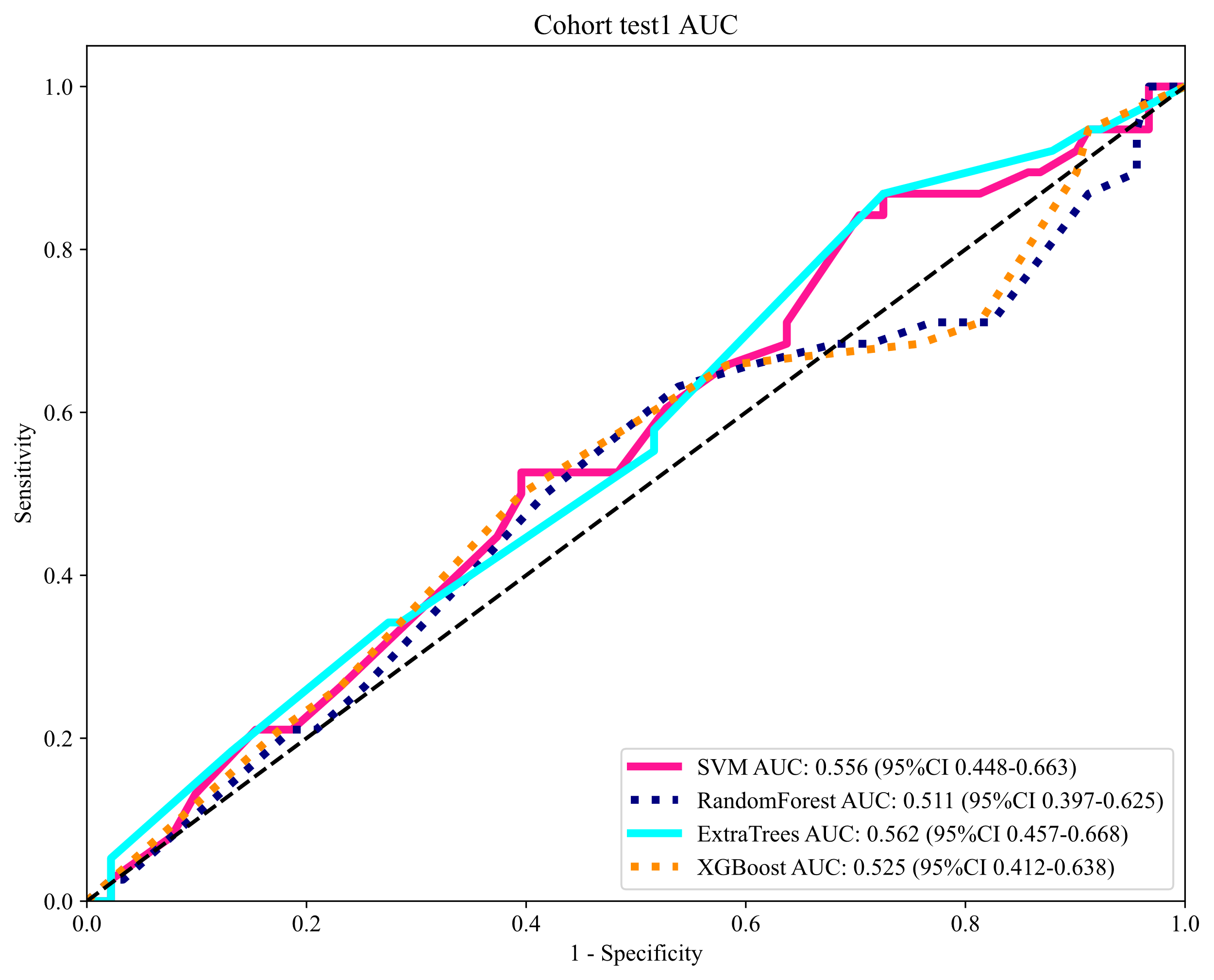

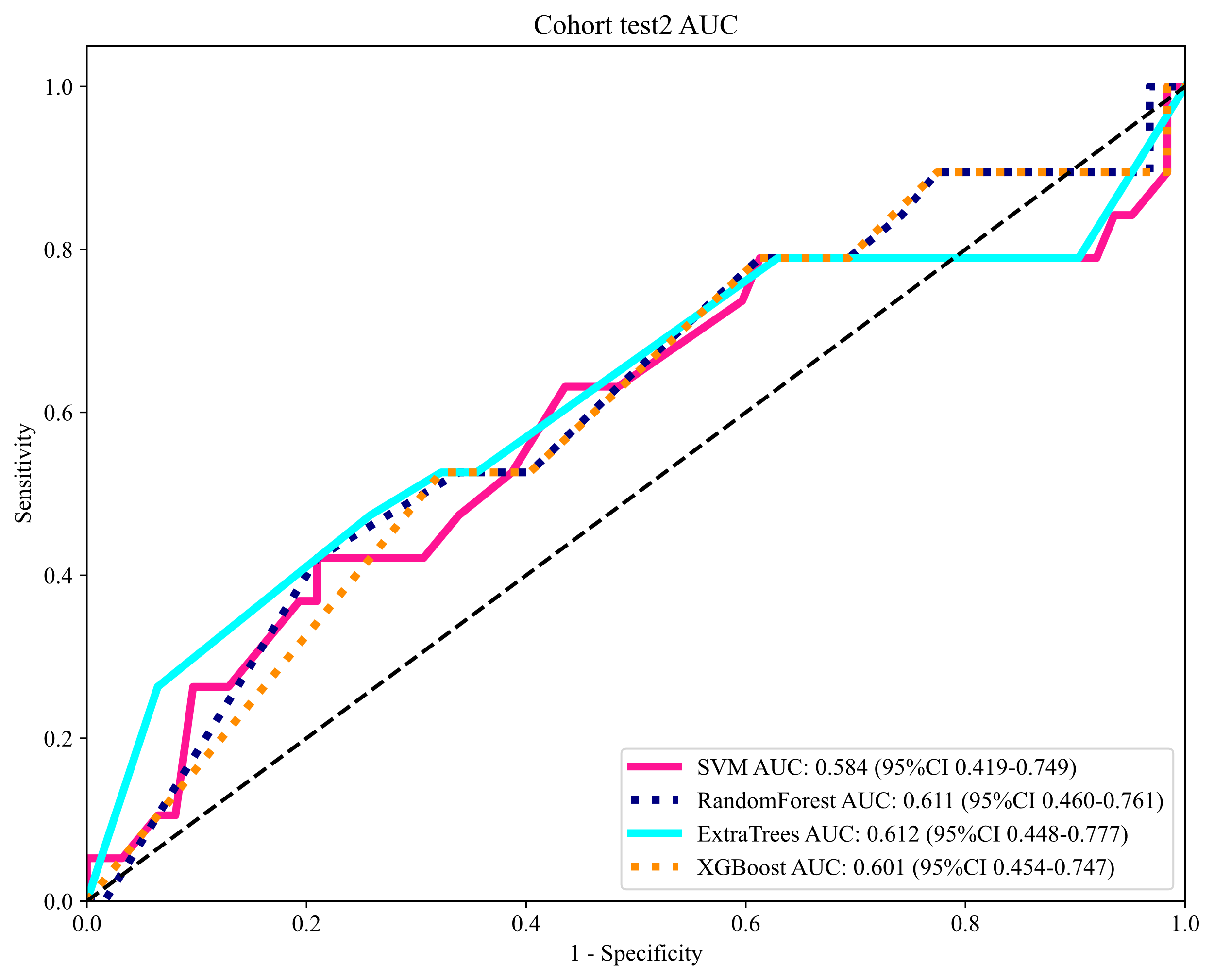


### 2B. Details of Radiomics Signature

| model_name | Accuracy | AUC | 95% CI | Sensitivity | Specificity | PPV | NPV | Cohort |
| --- | --- | --- | --- | --- | --- | --- | --- | --- |
| SVM | 0.935 | 0.970 | 0.934 - 1.000 | 0.931 | 0.936 | 0.844 | 0.973 | train |
| SVM | 0.787 | 0.825 | 0.704 - 0.946 | 0.864 | 0.720 | 0.731 | 0.857 | val |
| SVM | 0.713 | 0.776 | 0.699 - 0.854 | 0.921 | 0.626 | 0.507 | 0.950 | test1 |
| SVM | 0.568 | 0.612 | 0.467 - 0.757 | 0.737 | 0.516 | 0.318 | 0.865 | test2 |
| RandomForest | 0.804 | 0.863 | 0.787 - 0.939 | 0.793 | 0.808 | 0.605 | 0.913 | train |
| RandomForest | 0.766 | 0.745 | 0.598 - 0.892 | 0.636 | 0.880 | 0.824 | 0.733 | val |
| RandomForest | 0.798 | 0.795 | 0.713 - 0.877 | 0.711 | 0.835 | 0.643 | 0.874 | test1 |
| RandomForest | 0.691 | 0.781 | 0.690 - 0.873 | 1.000 | 0.597 | 0.432 | 1.000 | test2 |
| ExtraTrees | 0.888 | 0.941 | 0.890 - 0.993 | 0.897 | 0.885 | 0.743 | 0.958 | train |
| ExtraTrees | 0.809 | 0.845 | 0.732 - 0.957 | 0.909 | 0.720 | 0.741 | 0.900 | val |
| ExtraTrees | 0.744 | 0.796 | 0.712 - 0.880 | 0.684 | 0.769 | 0.553 | 0.854 | test1 |
| ExtraTrees | 0.802 | 0.729 | 0.570 - 0.888 | 0.737 | 0.823 | 0.560 | 0.911 | test2 |
| XGBoost | 0.832 | 0.836 | 0.752 - 0.920 | 0.862 | 0.821 | 0.641 | 0.941 | train |
| XGBoost | 0.766 | 0.760 | 0.628 - 0.892 | 0.727 | 0.800 | 0.762 | 0.769 | val |
| XGBoost | 0.643 | 0.749 | 0.668 - 0.831 | 0.921 | 0.527 | 0.449 | 0.941 | test1 |
| XGBoost | 0.667 | 0.747 | 0.634 - 0.860 | 0.842 | 0.613 | 0.400 | 0.927 | test2 |

Table 1. Model Performance of Different Machine Learning Algorithms in the Training, Validation, and Testing Sets.


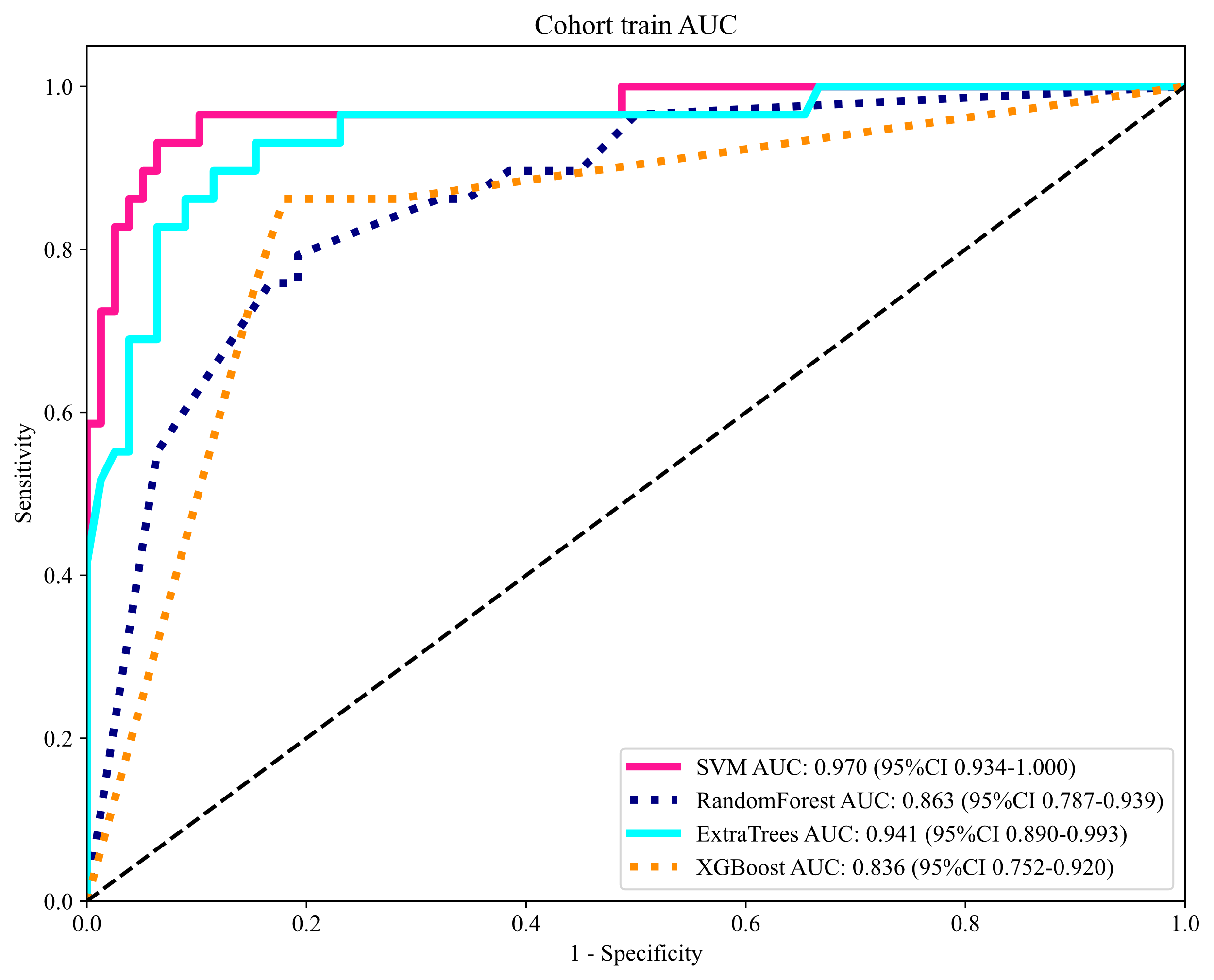

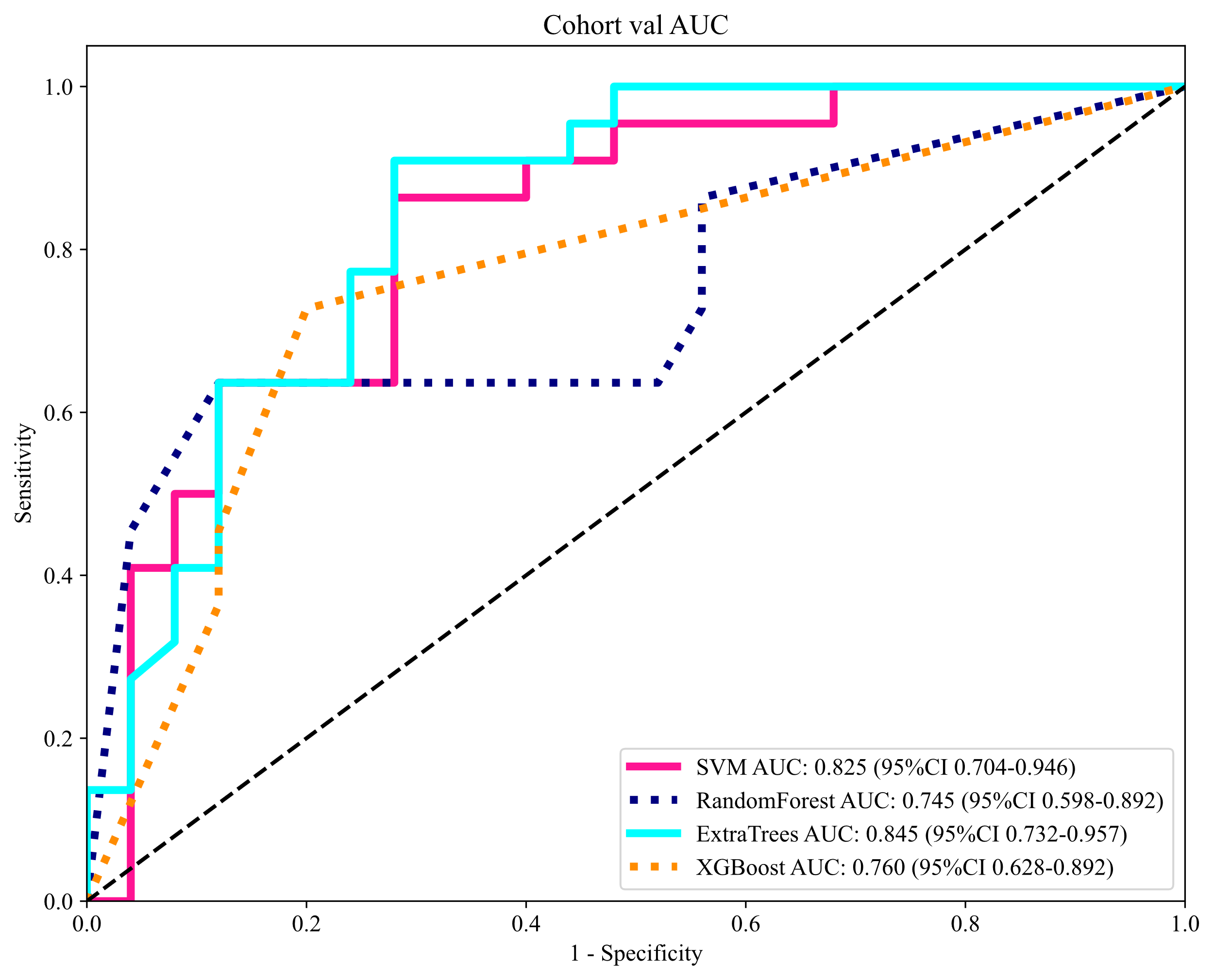

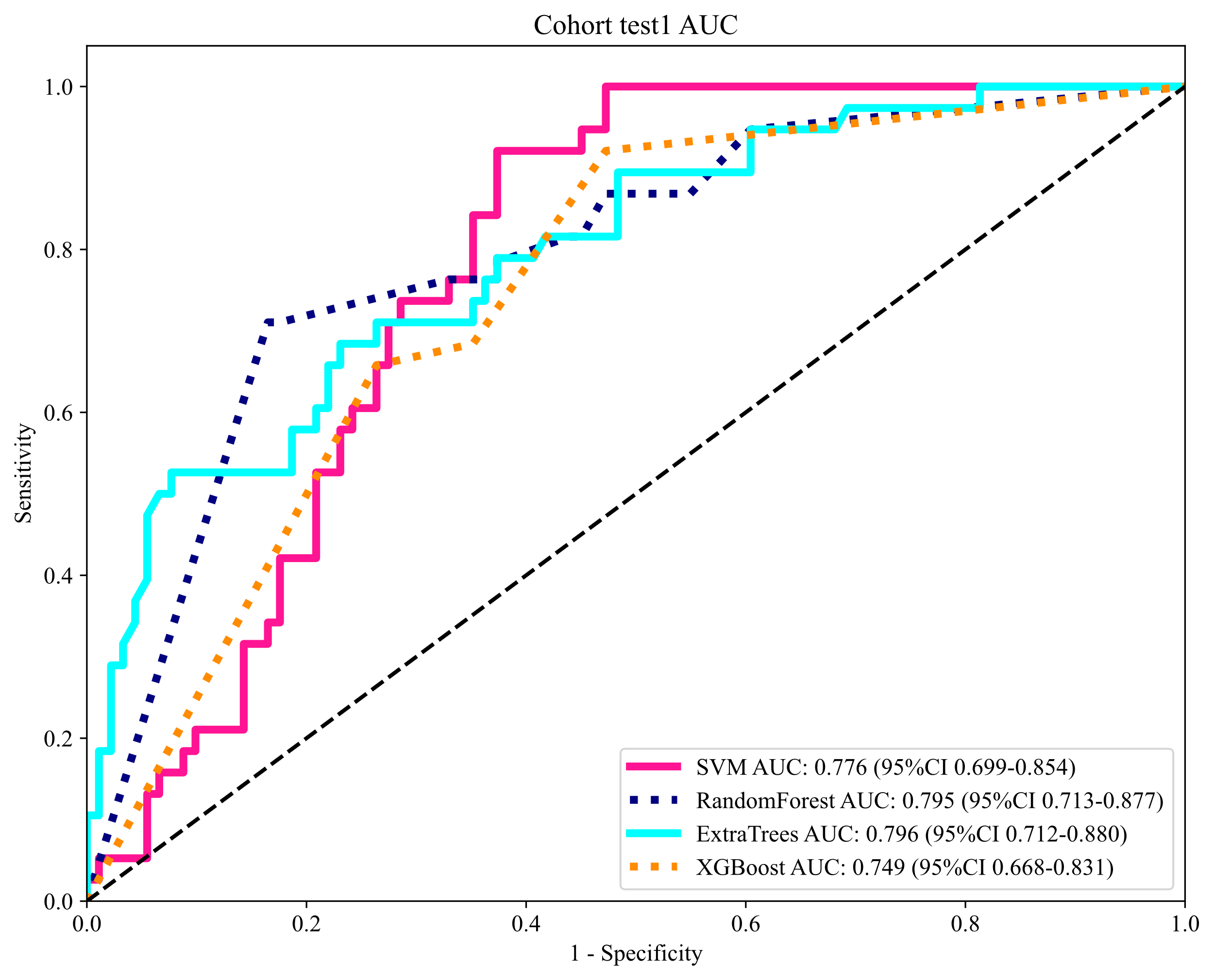

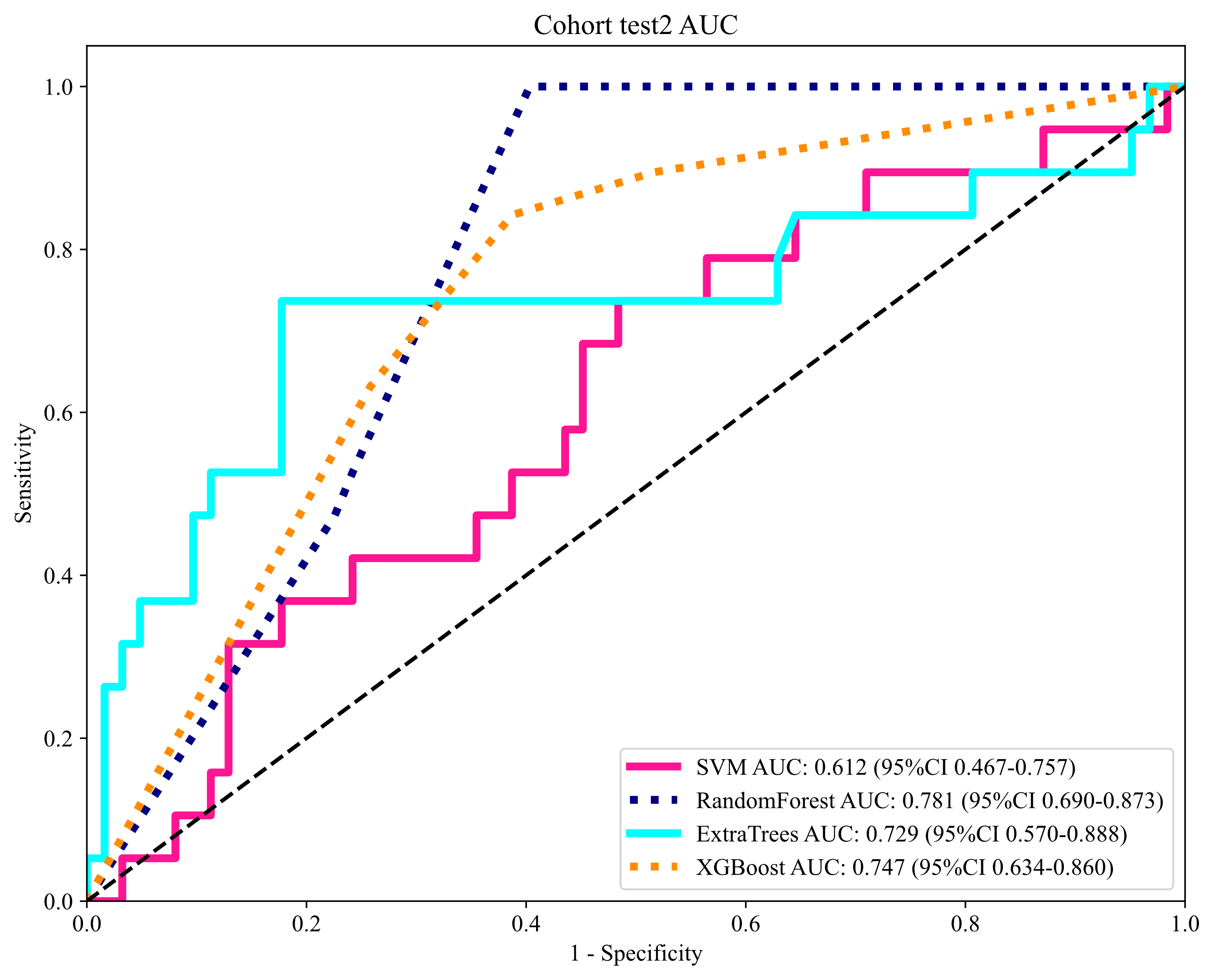


Figure 1. ROC Curves of Different Models in the Training, Validation, and Testing Sets.
